# Meeting need vs. sharing the market: a systematic review of methods to measure the use of private sector family planning and childbirth services in sub-Saharan Africa

**DOI:** 10.1186/s12913-018-3514-y

**Published:** 2018-09-10

**Authors:** Mardieh L. Dennis, Lenka Benova, Onikepe O. Owolabi, Oona M. R. Campbell

**Affiliations:** 1 Faculty of Epidemiology & Population Health, London School of Hygiene & Tropical Medicine, Keppel Street, London, WC1E 7HT UK; 2 Guttmacher Institute, 125 Maiden Lane 7th Floor, New York, NY 10038 USA

**Keywords:** Private sector, Family planning, Maternal health, Systematic review, Africa

## Abstract

**Background:**

Ensuring universal access to maternal and reproductive health services is critical to the success of global efforts to reduce poverty and inequality. Engaging private providers has been proposed as a strategy for increasing access to healthcare in low- and middle-income countries; however, little consensus exists on how to estimate the extent of private sector use. Using research from sub-Saharan Africa, this study systematically compares and critiques quantitative measures of private sector family planning and childbirth service use and synthesizes evidence on the role of the private sector in the region.

**Methods:**

We conducted a systematic review of the Medline, Global Health, and Popline databases. All studies that estimated use of private sector of family planning or childbirth services in one or more sub-Saharan African countries were included in this review. For each study, we extracted data on the key study outcomes and information on the methods used to estimate private sector use.

**Results:**

Fifty-three papers met our inclusion criteria; 31 provided outcomes on family planning, and 26 provided childbirth service outcomes. We found substantial methodological variation between studies; for instance, while some reported on service use from any private sector source, others distinguished private sector providers either by their profit orientation or position within or outside the formal medical sector. Additionally, studies measured the use of private sector services differently, with some estimating the proportion of need met by the private sector and others examining the sector’s share among the market of service users. Overall, the estimates suggest that the private sector makes up a considerable portion (> 20%) of the market for family planning and childbirth care, but its role in meeting women’s need for these services is fairly low (< 10%).

**Conclusions:**

Many studies have examined the extent of private sector family planning and childbirth service provision; however, inconsistent methodologies make it difficult to compare results across studies and contexts. Policymakers should consider the implications of both private market share and coverage estimates, and be cautious in interpreting data on the scale of private sector health service provision without a clear understanding of the methodology.

**Electronic supplementary material:**

The online version of this article (10.1186/s12913-018-3514-y) contains supplementary material, which is available to authorized users.

## Background

As the international development community shifts its focus from the Millennium Development Goals to the Sustainable Development Goals, universal access to maternal and reproductive health services remains critical to the global strategy for poverty and inequality reduction [1, 2]. Many low- and middle-income country governments have rolled out strategies to increase supply of and demand for public sector family planning and childbirth services [3–8]. However, some argue that reliance on the public sector alone to expand access to health services is impractical and that harnessing the contribution of private, non-government actors is the key to achieving universal healthcare coverage in low- and middle-income settings [9–11]. Proponents of publicly-financed health services, on the other hand, argue that encouraging growth of the private health sector is likely to exacerbate inequalities in access to care by making services financially unattainable for the poor [9, 12, 13].

Understanding non-government actors’ current contribution to health service provision is critical for determining if, how, and in which contexts to engage the private sector. While many studies have attempted to quantify the contribution of the private sector in low- and middle-income country (LMIC) contexts, there has been relatively little discussion of the philosophical and methodological considerations of doing so. One major challenge is defining what constitutes the “public” and “private” sectors. While sector is often defined in terms of the ownership or management of a health facility and dichotomized as public versus private, past research on health systems in LMICs has acknowledged that formalized partnerships between government-owned and non-government entities, government financing of private providers, and the practice of providers offering services in both government and privately-operated facilities have resulted in challenges in distinguishing the two sectors [14, 15]. Additionally, researchers of organizational theory argue that this public-private dichotomy does not adequately capture the range of factors that determine the degree to which a health facility or organization is publicly-oriented, and that health organizations should, instead, be conceptualized along a multi-dimensional continuum including ownership, financing, and mission. [16, 17]. These more nuanced definitions of sector, however, require details about health providers that are often not available or infeasible to collect in population-level assessments of the use of providers in different sectors.

Using an ownership-based definition of sector, private providers are believed to provide a substantial portion of maternal and reproductive health services in low- and middle-income countries; however, estimates of their role seem to vary considerably between studies and contexts [18, 19]. For instance, one recent study using Demographic and Health Survey (DHS) data reported that 38% of modern family planning users and in sub-Saharan Africa sought care in the private sector, while another recent study, also using DHS data, estimated this figure at 28% [20, 21]. Though some of the variation between the two estimates is due to different countries being included in the analyses, inconsistencies in how these percentages were calculated also had an effect.

Differences in measurement approaches increase the likelihood of researchers over- or underestimating the role of the private sector in provision of family planning and childbirth services. Using research from sub-Saharan Africa, this review has two main objectives: (1) to systematically compare and critique quantitative measures of private sector family planning and childbirth service use and (2) to descriptively synthesize evidence of the contribution of the private sector family planning and childbirth service use in the region. Further, by examining both an outpatient service largely requiring low- to mid-level clinical skills (family planning) and an inpatient service requiring mid- to high-level clinical skills (childbirth care), this study will highlight how the identified methodological approaches affect private sector use estimates for services delivered through different channels of the health system.

## Methods

### Scope of review

For the purposes of this review, we considered the private sector to encompass all providers owned by non-government actors. Given the descriptive nature of our outcomes of interest, peer-reviewed and grey literature papers of any study design were eligible for inclusion. We did not apply any restrictions on language or date of publication. For each study, we summarized the methods used to measure private sector service provision and the estimates reported. We discussed the strengths and weaknesses of the methods used in each study and how they might have biased the findings. This review is not registered and does not have a published protocol. Additional file 1 contains a Preferred Reporting Items for Systematic Reviews and Meta-Analyses (PRISMA) checklist for this review.

### Search strategy

**Fig. 1:**
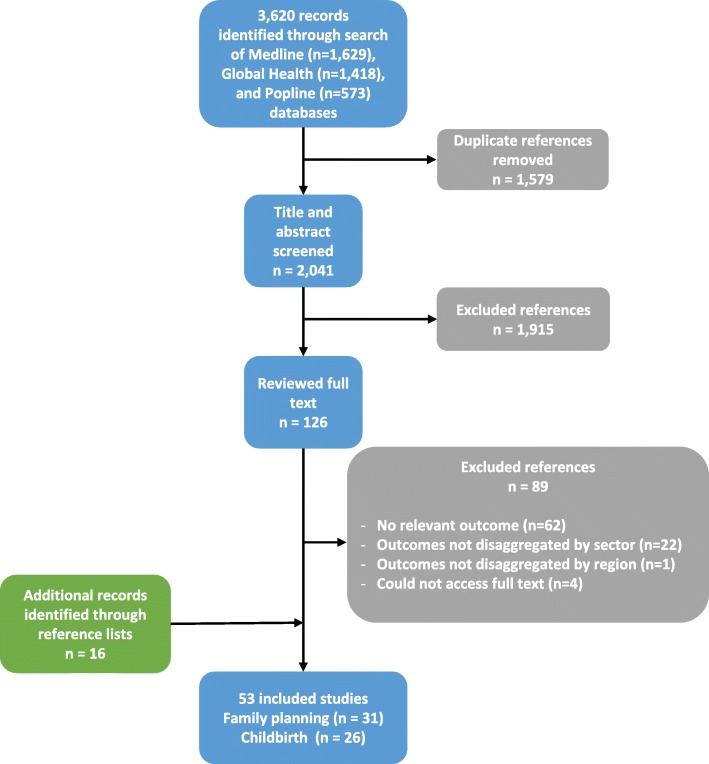
PRISMA flow diagram

We identified studies by searching the Medline, Global Health, and Popline databases, using a combination of keywords and MeSH terms covering the following broad themes: (1) sub-Saharan Africa, (2) contraception or childbirth services, and (3) private sector. Additional file 2 contains the full list of keywords and MeSH terms used. We conducted our search on October 26, 2016. A total of 3,620 records were identified from the three databases and imported into Covidence, an online systematic review management platform. After removing duplicates, the titles and abstracts of 2,041 publications were screened for inclusion in the review. Publications clearly outside of the scope of the review, covering topics unrelated to use of family planning or childbirth services in sub-Saharan Africa, were excluded at this stage, while the 126 studies that appeared definitely or potentially relevant were selected for full text screening. MLD and OOO screened the studies at each stage, and any discrepancies were discussed and resolved. Studies that did not present private sector use estimates for family planning or childbirth care, or that combined figures for multiple services, were excluded. Studies that did not present outcomes for at least one sub-Saharan African country, or presented estimates from sub-Saharan Africa pooled with those from other regions, were also excluded. We selected 37 studies for inclusion in the review; 16 additional papers were identified through systematically scanning the references of all included studies (Fig. 1). MLD extracted information on the included studies’ methods and results for this analysis.

## Results

### Overview of included studies

**Fig. 2:**
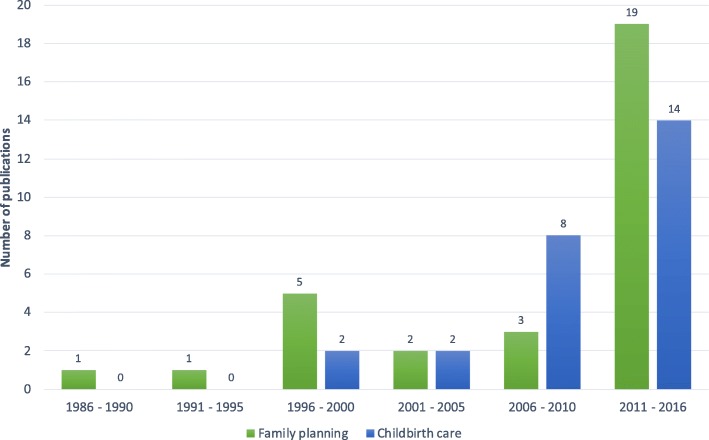
Number of included studies by publication date

Fifty-three papers met our inclusion criteria; 31 included outcomes on family planning, while 26 provided outcomes on childbirth services (Additional file 3). Studies on private sector provision of family planning and childbirth services in sub-Saharan Africa have proliferated in recent years, with the number of included papers published during the 6-year period from 2011 to 2016 exceeding the number published over the preceding 25 years combined (Fig. 2).

The included papers provided estimates of private sector family planning or childbirth service use for 40 countries in sub-Saharan Africa over a 30-year period from 1984 to 2014. Certain countries, such as Ghana, Kenya, and Zimbabwe, were studied extensively over time while others, such as the Gambia and Somalia, were not studied at all (Additional file 4).

More than half of the included studies focused on a single country (*n* = 31); the remaining studies included multi-country comparative analyses of two to 36 countries. The majority of studies looked at cross-sectional data at one point in time (*n* = 40), while 13 studies examined trends over time using repeated cross-sectional data [21–33].

### Measuring use of private sector family planning & childbirth services

#### Data sources

Forty-seven of the 53 studies used household survey data to estimate use of private sector family planning and/or childbirth services; the majority of these studies used the Demographic and Health Surveys (*n* = 27), while others used data from demographic surveillance sites [34, 35], national maternal health surveys [36], or other smaller, sub-national surveys [23, 24, 37–50]. Three studies conducted surveys that sampled women at a health facility [51], market [52], or through respondent-driven sampling [53]. The remaining three studies used routine health service statistics to estimate the proportion of facility births that occurred within the private sector [54–56].

#### Source of care: Defining the private sector

**Fig. 3:**
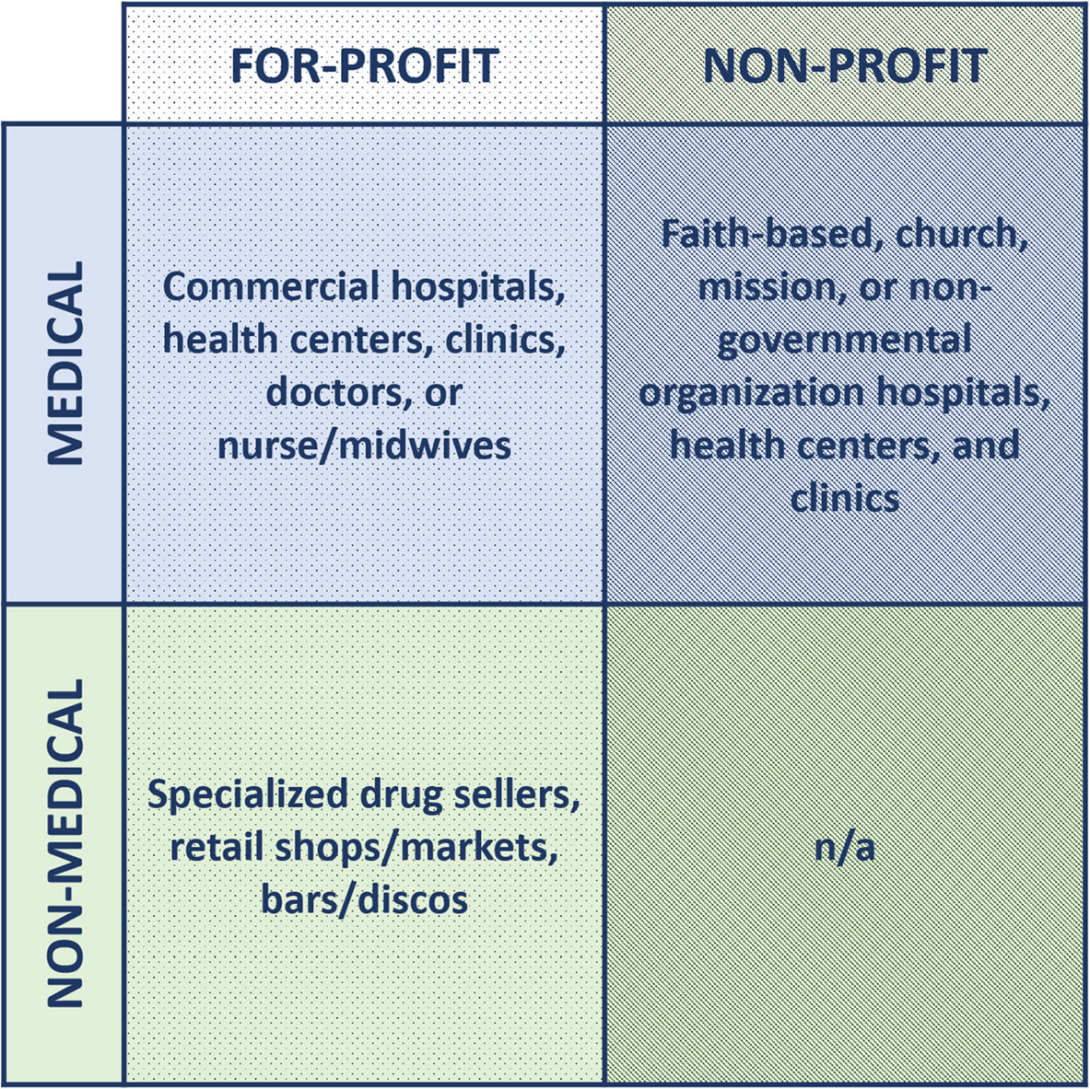
Classification of private sector providers

The studies included in this review contained over 40 unique terms to describe private sector sources of family planning and childbirth services (Additional file 5). Throughout the literature, the “private sector” referred to a range of for-profit, not-for-profit, faith-based, medical, and informal providers that were managed by non-government actors. While some studies reported on service use from any private sector source, others distinguished private sector providers by two key characteristics: (a) their commercialization or profit orientation and/or (b) their position within or outside of the medical sector (Fig. 3). Among the included studies, the private for-profit sector included both medical and non-medical providers, while the private non-profit sector seemed to refer exclusively to medical providers. Additional file 6 displays the frequency with which each unique private sector term appeared in the included studies, categorized by profit orientation.

#### Populations under study: Coverage vs. market share

**Fig. 4:**
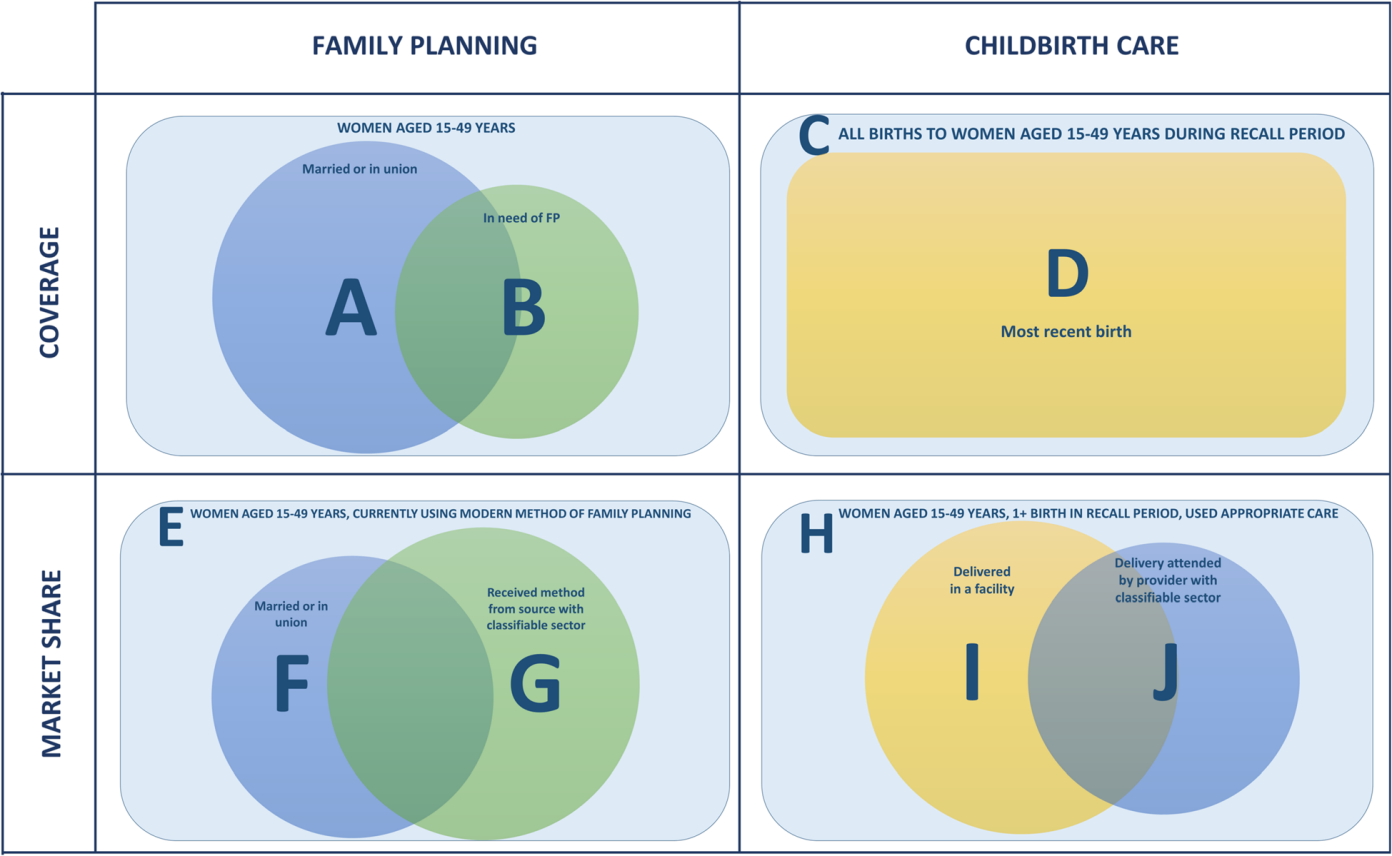
Denominators for measuring private sector coverage and market share in included studies

The studies included in this review examined private sector use within two general population groups: (1) women in need or “at risk” of needing family planning or childbirth services and (2) users of those services. We used the term private sector *coverage* to indicate the proportion of women in need who were using family planning or childbirth services from a private sector source, or the proportion of health service need met by the private sector. Private sector *market share*, on the other hand, refers to the proportion of family planning or childbirth service users who received care from a private sector source. Of the 31 studies that examined use of private sector family planning services, five reported coverage estimates [20, 21, 57–59], 30 reported market share estimates, and four reported both market share and coverage estimates [20, 21, 57, 59] (Additional file 6). For childbirth services, 22 of 26 papers presented private sector coverage estimates, seven reported on private sector market share [35, 52, 54–57, 60], and three reported both market share and coverage estimates [35, 57, 61] (Additional file 6). Although we grouped these outcomes into two broad categories, there was substantial variation within each category in how these populations were defined. For instance, three studies considered the population in need of family planning to be all women married or in union (regardless of fertility preferences or desires) [21, 58, 59], while two studies reported use among all women with d need for family planning (regardless of marital status) [20, 57], following the most recent consensus definition of need for contraception [62]. We summarized the different populations used to examine private sector coverage and market share in Fig. 4.

#### Unit of analysis

There were differences between studies in the unit of analysis for examining source of family planning and childbirth services. Of the 31 studies that reported on use of private sector family planning services, 13 described source of care during women’s most recent supply of contraceptives [20, 22, 24–26, 29, 31, 41, 50, 53, 58, 59, 63] and one described where women obtained care when they first started using their current method [40]. The majority of studies (*n* = 17), however, did not state their unit of analysis [19, 21, 27, 28, 38, 39, 42, 44, 45, 51, 57, 64–68].

When describing use of childbirth services, the included studies generally adopted either a birth-based (*n* = 6) [23, 54–56, 69, 70] or a woman-based approach (*n* = 13) [24, 30, 36, 37, 43, 46–50, 57, 60, 68]. The unit of analysis for birth-based approaches was all births that occurred over the study recall or review period. The woman-based approach, on the other hand, included only one birth per woman, and all of the included studies adopting this approach used a woman’s most recent birth as the unit of analysis. One study used a hybrid approach, taking information about all of a woman’s births and categorized her according to where she sought care across births [32]. Six of the 26 studies that reported on use of private sector childbirth services did not state their unit of analysis [34, 35, 52, 71–73].

#### Treatment of missing information

Although missing information on source of care and family planning and childbirth service use and need has the potential to bias estimates, relatively few studies described how they treated such missing information.

Of the studies that examined private sector market share for family planning and childbirth services, only 9 out of 30 [20, 24, 25, 27, 40, 42, 57, 63, 67] and two out of seven [57, 60], respectively, indicated how missing data on source of care was treated. Among the studies that did provide this information, the convention was to either include women with missing information as part of the market, but report their source of care as missing or unknown, or to exclude them from the market entirely.

Of the five studies that reported on private sector coverage of family planning services, only two described how they treated women with missing data on family planning need [20, 57] and three described how they treated women with missing data on source of care [20, 57, 58]. In those studies, women with incomplete information on family planning need were considered to be not in need of contraception, while those with missing information on source of care were considered to not have received care in the private sector. For childbirth services, all reported deliveries were considered in need of care. Campbell et al. [8] is the only study that discussed missing information on delivery care need, and the authors found no missing data for that variable [57]. Six of the 22 studies that examined private sector coverage of childbirth services reported that women with missing information on source of care were considered to have not received care in the private sector [24, 35, 36, 43, 57, 60].

### Use of private sector family planning and childbirth services in sub-Saharan Africa

**Fig. 5:**
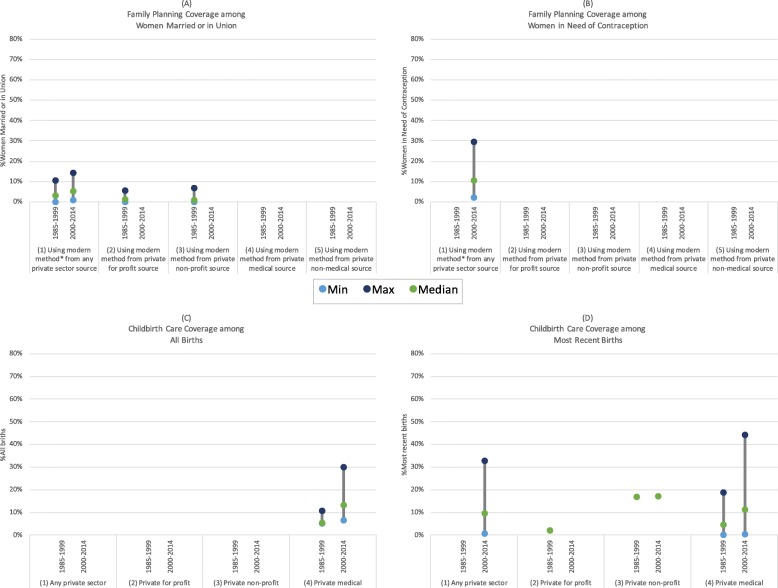
(**a**) Family planning coverage among women married or in union; (**b**) Family planning coverage among women in need of contraception; (**c**) Childbirth care coverage among all births; (**d**) Childbirth care coverage among most recent births

**Fig. 6:**
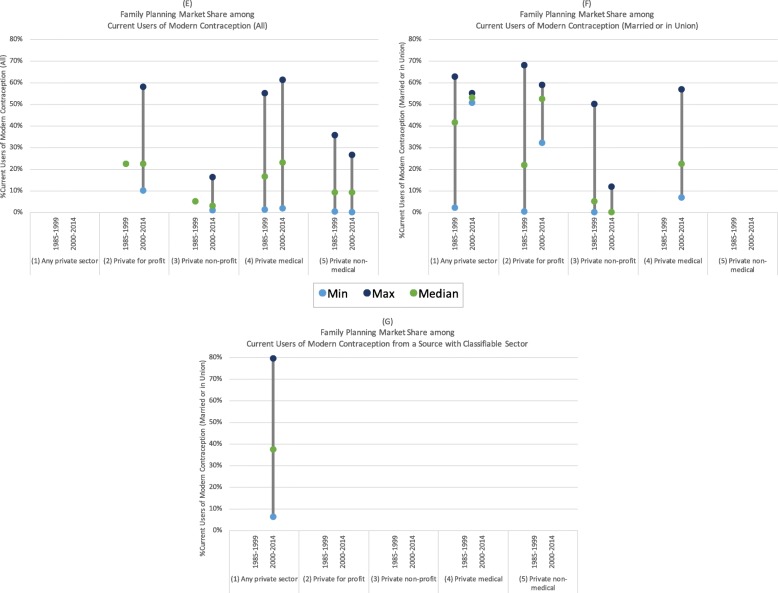
(**e**) Family planning market share among all current users of modern contraception; (**f**) Family planning market share among married/in union current users of modern contraception; (**g**) Family planning market share among all current users of modern contraception from a source with a classifiable sector

**Fig. 7:**
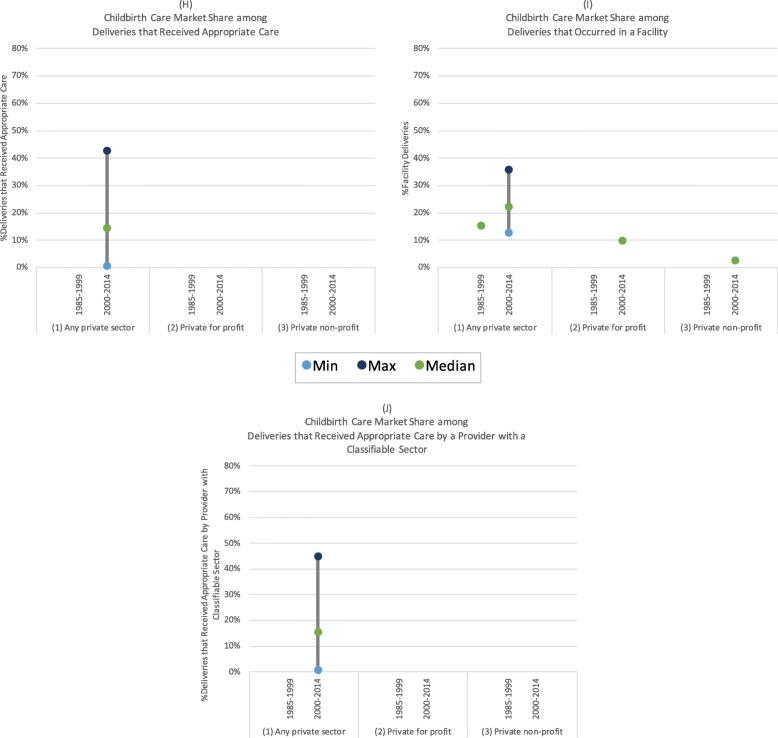
(**h**) Childbirth care market share among deliveries that received appropriate care; (**i**) Childbirth care market share among deliveries that occurred in a facility; (**j**) Childbirth care market share among deliveries that received appropriate care by a provider with a classifiable sector

Given the differences in how use of private sector family planning and childbirth services were defined and calculated, as well as the many settings and periods in which these studies took place, we observed considerable heterogeneity in the estimates of our key outcomes of interest (Additional file 6). To assess trends in outcomes for the region as a whole, and to highlight the influence of methodological differences, we summarized the minimum, maximum, and median national and sub-national estimates of private sector coverage and market share for family planning and childbirth services in sub-Saharan Africa by period under study in Figs. 5, 6 and 7. Aggregated regional estimates are not represented in the figures. To facilitate comparisons between studies, we only included those that provided coverage or market share estimates representing at least one of the following private sector provider classifications: (1) all private sector, (2) private for profit, (3) private non-profit, (4) private medical, or (5) private non-medical. For family planning studies, we only included estimates for private sector market share and coverage for all modern methods; estimates for individual methods or that included traditional methods were excluded.

#### Family planning coverage

Ugaz et al. [21] estimated that private sector coverage among women married or in union was relatively low in the sub-Saharan Africa region, ranging between 3 and 6% from 1992 to 2012. Looking exclusively at the population of women in need of contraception, regardless of marital status, Campbell et al. [20, 57] estimated higher private sector coverage, with 14% private sector coverage of modern contraceptive need. Similarly, comparing Fig. 5a and b, we observed that estimates of private sector family planning coverage tended to be higher among women in need compared to all women married or in union [20, 21, 58, 59]. As expected, the proportions of women using modern methods from private non-profit or for-profit providers was smaller than the proportion of women using modern methods from any private sector source.

#### Childbirth service coverage

Benova et al. (2015) estimated that 10% of women in sub-Saharan Africa delivered in the private sector for their most recent birth, either at a private facility or in a non-facility location with a private medical provider, while Wodon et al. (2012) generated a lower estimate, at 6.8% of women [61, 68]. Yoong et al. [70] estimated that an average of 7.7% of all births in the region received childbirth care from a private medical provider, specifically in a private medical facility. In Fig. 5c and d, estimates of private sector coverage appeared to be quite similar and increasing between the 1985–1999 and 2000–2014 periods, with median values increasing from 5 to 14 and 4 to 11% among all births and most recent births, respectively [23, 24, 30, 36, 46, 48, 50, 68, 69]. Private non-profit provider coverage of most recent births was estimated around 17% in both periods, and private for profit provider coverage appeared negligible; however, the studies reporting on this outcome were conducted exclusively in rural areas in Kenya and Tanzania with access to a mission hospital [46, 49].

#### Family planning market share

We observed much greater heterogeneity in estimates of private sector market share estimates for sub-Saharan Africa compared to those of private sector coverage. Campbell et al. [8, 20, 57] estimated that 35% of all modern family planning users and 38% of modern family planning users who received their method from a source with a classifiable sector obtained care from a private sector provider. Figure 6g shows that the family planning market share among women who obtained care from a source with a classifiable sector ranged from 6% in Rwanda (2010) to 80% in Gabon (2012) [20].

Both Ugaz et al. [21] and Wodon et al. [68] estimated that approximately 28% of all modern family planning users in sub-Saharan Africa received their method from a private medical provider. This ranged from countries with less than 2% private medical market share (Burundi, 1987; Sao Tome and Principe, 2008/9) to countries with greater than 60% private medical market share (Democratic Republic of the Congo, 2007; Nigeria, 2008) (Fig. 6e) [50, 68, 74].

Among all users of modern contraception and those married or in union, private for-profit providers appeared to have a greater market share compared to private non-profit providers (Figs. 6e & 6f) [22, 29, 33, 41, 59, 66–68]. There also seemed to be greater use of private medical providers compared to non-medical providers among current users of modern contraception; however, this may be because Wodon et al. (2012) classified pharmacies as medical providers while others distinguished facilities from pharmacies or specialized drug sellers (Fig. 6e) [50, 67, 68].

#### Childbirth service market share

Only two studies comprehensively examined childbirth service market share across a large number of countries [57, 61]. Benova and colleagues estimated that 20% of women who gave birth under appropriate care conditions (in a facility or with a skilled-birth attendant) in sub-Saharan Africa received care in the private sector (Fig. 7h) [61]. Looking at source of care among women who received appropriate care from a provider with a classifiable sector increased this estimate slightly to 22% (Fig. 7j) [57, 61]. Three smaller studies from Kenya, South Africa, and Uganda examined private sector market share among facility births, and estimates ranged from 15% (South Africa, 1990) to 36% (Uganda, 2007) [35, 54, 55]. One study from Kenya found that private for- profit providers had a greater market share among facility births (10%) compared to private non-profit providers (3%) [54]. In contrast to private sector family planning market share, none of the estimates of private sector market share for childbirth services exceeded 45%.

## Discussion

We identified 53 papers that estimated use of private sector family planning and childbirth services in sub-Saharan Africa. Consistent with beliefs about the private sector’s role in the delivery of healthcare in low- and middle-income countries more generally, our findings suggest that in many African nations, the private sector provides a substantial proportion of both family planning and childbirth services among service users [18, 19]. However, among women in need of these services, private sector coverage is comparatively low. Further, these results suggest that the private sector provided more family planning services than childbirth care in the region. This is due to the less specialized nature of certain family planning methods such as condoms, which allow for provision of services by lower-skilled drug sellers and commercial shops. Although the included studies provided estimates for a majority of countries in the region, it is important to acknowledge that some countries were not studied or have estimates that are outdated or not representative at the national level.

More revealing, however, are our findings on the lack of consistency with which researchers defined the private sector and measured its use. While there is clear heterogeneity between countries in the actual role of the private sector, methodological differences also have the potential to greatly affect estimates of private sector participation. It is therefore important to understand the strengths and weaknesses of each analytical approach when interpreting findings.

When it comes to defining the private sector, a more inclusive definition naturally yields a higher estimate. The extent to which including or excluding certain segments of the private sector biases an outcome depends both on context and the service being examined. For instance, while private non-medical providers can conceivably provide a number of modern family planning methods such as condoms or pills, appropriate delivery care should, according to World Health Organization recommendations, occur with a skilled health provider, namely a midwife or doctor [75]. Thus, only examining private medical provision of services is likely to present an incomplete picture of the role of the private sector in delivering family planning services, but a more accurate picture for appropriate childbirth care. As has been noted elsewhere, non-profit and faith-based services are often provided in collaboration with governments and therefore may be difficult to distinguish from public sector care, particularly when relying on women providing self-recall survey data [14, 15, 76–78]. Estimates of all private sector and private non-profit sector service provision are therefore likely to underestimate their true contributions.

Selecting which population to study also requires careful consideration. Examining use of the private sector within a broader population tends to yield lower estimates compared to use among a more narrowly defined population group. As a result, coverage estimates are always equal to or less than market share. In contexts where use of a service is universal or very high within a population, coverage will be equal or similar to, but lower than, market share. In contexts where use of a service is moderate or low, coverage will be much lower than market share.

Because private sector coverage is bounded by total use of a service, comparing private sector coverage estimates between countries with very different levels of total use is challenging. For example, a country (A) with very high use of family planning services, but very low use of the private sector among users, might have the same absolute private sector coverage as a country (B) with low use of family planning services, but very high use of the private sector among users. In such a case, examining coverage alone would lead to the conclusion that the private sector plays a similar role in service provision in each country; however, looking at market share would reveal very different dynamics at play. Similarly, looking at market share alone might lead one to conclude that the private sector serves a greater proportion of the population in country B than in country A, whereas coverage estimates would indicate that the share of total need satisfied by the private sector is similar in both countries.

Population selection also has important implications on estimates within the categories of coverage and market share. Researchers frequently measure private sector family planning coverage as the use of modern contraception from a private sector source among women married or in union, and less frequently among all women in need of contraception. To estimate the latter requires including women who are sexually active but not in union, and excluding women not in need of contraception because they are pregnant or because they wish to have more children in the near future. For secondary analysis of survey data with limited information on fertility preferences and need for contraception, examining use of private sector services among married women might be a reasonable approach. However, this will certainly underestimate private sector coverage given that some proportion of the married population desire to become pregnant and are therefore not in need of contraception. The papers included in this review have also looked at market share among all current users of modern contraception and current users who are married or in union only. Given that service use by married women might not represent the population of women in need, it is preferable to look at source of care for all current users, unless the purpose of the analysis is to compare the experiences of married women to the general population or to unmarried women.

Among papers that looked at private sector family planning market share among all users of modern contraception, regardless of marital status, some limited analysis to women who received care in the private or public sectors. Excluding women who received care from a source whose sector could not be classified from the population under study leads to slightly higher estimates of private sector market share; the extent of overestimation depends on the size of the “unknown sector”.

Another consideration when estimating private sector family planning market share is whether to examine source of care when a woman most recently received her current method or when she first received the method. While most papers in this review examined most recent source, it might also be important to understand where women went to start and whether they switch the source of their current family planning method.

For private sector childbirth care coverage estimates, researchers generally used a birth-based approach, looking at use of private sector childbirth services among all births that occurred during a given period, or a woman-based approach, examining source of care for a woman’s most recent birth. Analyzing all births allows for a larger sample size and better represents births that occurred within a given period among women with both short and long birth intervals. Analyzing private sector childbirth care use among most recent births only, on the other hand, will over-represent births to women with longer birth intervals. As women with short birth intervals are often less likely to deliver in a facility or have a skilled attendant at birth [79, 80], private sector coverage among all births is likely to be lower than among coverage for most recent births only. Nevertheless, estimates using all births and those using the most recent birth appear similar; suggesting neither approach greatly affects the conclusions about source of care.

Some studies that examined private sector market share for childbirth services specifically looked at the use of the private sector among women who received appropriate delivery care, defined as either by a skilled birth attendant or in a health facility. Estimating the market share among facility births only excludes provision of care at home or in another non-facility setting by a private medical provider, and therefore may underestimate private sector market share for childbirth services. However, in contexts where home deliveries with a skilled birth attendant are rare, looking exclusively at facility births is unlikely to greatly affect private sector childbirth service market share estimates. As with family planning market share, focusing solely on use of the private sector among users of childbirth services from providers with a classifiable sector generated a slightly greater estimated private sector childbirth care market share compared to analysis of use among all users of appropriate childbirth services.

Although missing data on need for services and source of care has the potential to impact estimates of private sector provision of family planning and childbirth services, relatively few papers in this review discussed the extent or treatment of missing data. To ensure that findings can be clearly interpreted, it is important for researchers to acknowledge and describe the effects of missing information on their outcomes.

On a more practical level, data collection methods also influence the type of private sector use outcome that can be estimated. As private sector coverage requires information on the number of women in need who do not seek care, it can only be measured through population surveys. Facility records can be used to estimate private sector market share among facility births if private sector facilities report births, and this would approximate private sector market share for childbirth services in settings where home births with skilled attendants are uncommon. If health facility records are accurate and women tend to seek care within their catchment area, this may be more cost effective than population surveys for estimating private sector childbirth service market share for a given geographic region. Considering the wide range of private sector medical and non-medical outlets through which modern methods of family planning can be accessed, it would be much more difficult to ascertain private sector family planning market share through facility records.

## Conclusion

Our review suggests that the private sector plays a substantial role in the delivery of family planning and childbirth services in sub-Saharan Africa. Interest in the role of private sector provision of health services in low- and middle-income countries continues to grow; however, there appears to be lack of consensus on how to appropriately measure and report the use of private sector services. Though a plethora of studies have examined the role of the private sector in providing family planning and childbirth services in sub-Saharan Africa, inconsistencies in how researchers define the private sector and measure its use make it difficult to compare results across studies and contexts. Slight changes in methodology can have substantial impact on private sector service provision outcomes. To ensure correct interpretation of findings, it is therefore imperative that researchers better describe their methods and acknowledge the potential biases in their analytical approaches. Additionally, national- and regional-level policymakers should consider the implications of both private market share and coverage estimates and take care in interpreting data on the scale of private sector health service provision without a clear understanding of the methodologies used.

## Additional files

Additional file 1: PRISMA checklist. (DOC 62 kb)

Additional file 2: Key Words & MeSH Terms for Literature Review. (DOCX 104 kb)

Additional file 3: Descriptive summary of studies included in review. (DOCX 46 kb)

Additional file 4: Studies on private sector family planning or childbirth care use by country & period. (PDF 257 kb)

Additional file 5: Frequency of terms used to describe private sector sources in included studies. (PNG 336 kb)

Additional file 6: Included study methods and results summary. (DOCX 121 kb)

## Abbreviations

DHS: Demographic and Health Survey
LMIC: Low- and middle-income country
PRISMA: Preferred Reporting Items for Systematic Reviews and Meta-Analyses
