# Supplementary material for: Meeting need vs. sharing the market: a systematic review of methods to measure the use of private sector family planning and childbirth services in sub-Saharan Africa
Source: BMC Health Serv Res. 2018 Sep 10;18:699. doi: 10.1186/s12913-018-3514-y (PMC6131793; doi:10.1186/s12913-018-3514-y)
Supplement: Supplementary file 2 — Key Words & MeSH Terms for Literature Review. (DOCX 104 kb) [file 12913_2018_3514_MOESM2_ESM.docx]

### Key Words & MeSH Terms for Literature Review

#### Medline Search Strategy

Search 1: Sub-Saharan Africa Terms

| exp Africa South of the Sahara/ or |
| --- |
| Sub-Saharan Africa* or |
| Benin or Burkina Faso or Burundi or "Central African Republic" or Chad or Comoros or Eritrea or Ethiopia or Gambia or Gabon or Guinea-Bissau or Liberia or Madagascar or |
| Guinea not (New Guinea or Guinea Pig* or Guinea Fowl) or |
| Congo adj2 (Democratic or Republic or Brazzaville or Kinshasa) or Zaire or |
| Malawi or Mali or Mozambique or Namibia or Rwanda or Sierra Leone or Somalia or South Sudan or Tanzania or Togo or Uganda or Zimbabwe or Cape Verde or Cabo Verde or Cameroon or Cameroun or Cote d'Ivoire or Ivory Coast or Djibouti or Ghana or Kenya or Lesotho or Nigeria or Sao Tome or Senegal or Sudan or Swaziland or Zambia or |
| Niger not (Aspergillus or Aspergilus or Peptococcus or Schizothorax or Cruciferae or Gobius or Lasius or Agelastes or Melanosuchus or radish or Parastromateus or Orius or Apergillus or Parastromateus or Stomoxys or Hyoscyamus or Cephalophus or Pterostichus) or |
| (multi#country or countries or multi#country or multi#level or ecological).m_titl. |

Search 2: Family Planning Terms

| exp Family Planning Services/ or |
| --- |
| exp Contraceptive Agents/ or |
| exp Contraception/ or |
| exp Reproductive Health/ or |
| family planning or birth control or contracepti* or |
| sterili#ation or vasectomy or tubal ligation or |
| IUD or IUCD or intrauterine adj2 (device or system) or |
| injectable* adj2 (hormon* or estrogen or oestrogen or progestogen) or Depoprovera or Depo-Provera or Depo Provera or Noristerat or |
| implant* adj2 (hormon* or contracepti*) or Implanon or Norplant or |
| exp Condoms/ or condom* or |
| pill adj3 (morning after or emergency or Levonorgestrel or hormon* or estrogen or oestrogen or progestogen) or |
| lactational adj2 (amenorrhea or amenorhoea) or |
| "Standard Days Method" or cycle beads or |
| unmet need or |
| met need |

Search 3: Delivery Care Terms

| exp Delivery, Obstetric/ or |
| --- |
| exp Maternal Health Services/ or |
| exp Cesarean Section/ or |
| exp Parturition/ or |
| delivery adj2 (child or obstetric) or |
| caesarean or cesarean or c-section or csection or |
| birth* adj2 (home or facility or child) or childbirth* or |
| maternal or maternity servic* |

Search 4: Private Sector Terms

| exp Private Sector/ or | |
| --- | --- |
| private adj2 (sector or for-profit or facilit* or provider* or clinic* or hospital* or pharmac* or drug seller*) or privati#ed or privati#ation or public-private or private or | |
| NGO or non-government* or nonprofit or not#for#profit or non#profit or non#for#profit or | |
| informal adj2 (sector or provider*) or traditional adj2 (healer* or doctor* or provider*) or | |
| exp Hospitals, Religious/ or | |
| exp Religious Missions/ or | |
| charit* or FBO or mission or faith#based or religious or faith-inspired or Christian or Catholic or muslim or Islam* or | |
| exp Social Marketing/ or | |
| social* market* or | |
| franchis* or |  |
| exp Contract Services/ or | |
| out or service* adj2 (contracting or contracted or contract) | |

Search 5: Final Search

1 AND (2 or 3) AND 4

*Global Health Search Strategy*

Search 1: Sub-Saharan Africa Terms

| exp "Africa South of Sahara"/ or |
| --- |
| Sub-Saharan Africa* or |
| Benin or Burkina Faso or Burundi or "Central African Republic" or Chad or Comoros or Eritrea or Ethiopia or Gambia or Gabon or Guinea-Bissau or Liberia or Madagascar or |
| Guinea not (New Guinea or Guinea Pig* or Guinea Fowl) or |
| Congo adj2 (Democratic or Republic or Brazzaville or Kinshasa) or Zaire or |
| Malawi or Mali or Mozambique or Namibia or Rwanda or Sierra Leone or Somalia or South Sudan or Tanzania or Togo or Uganda or Zimbabwe or Cape Verde or Cabo Verde or Cameroon or Cameroun or Cote d'Ivoire or Ivory Coast or Djibouti or Ghana or Kenya or Lesotho or Nigeria or Sao Tome or Senegal or Sudan or Swaziland or Zambia or |
| Niger not (Aspergillus or Aspergilus or Peptococcus or Schizothorax or Cruciferae or Gobius or Lasius or Agelastes or Melanosuchus or radish or Parastromateus or Orius or Apergillus or Parastromateus or Stomoxys or Hyoscyamus or Cephalophus or Pterostichus) or |
| (multi#country or countries or multi#country or multi#level or ecological).m_titl. |

Search 2: Family Planning Terms

| exp family planning/ or |
| --- |
| exp contraceptives/ or |
| exp contraception/ or |
| exp reproductive health/ or |
| family planning or birth control or contracepti* or |
| sterili#ation or vasectomy or tubal ligation or |
| IUD or IUCD or intrauterine adj2 (device or system) or |
| injectable* adj2 (hormon* or estrogen or oestrogen or progestogen) or Depoprovera or Depo-Provera or Depo Provera or Noristerat or |
| implant* adj2 (hormon* or contracepti*) or Implanon or Norplant or |
| exp Condoms/ or condom* or |
| pill adj3 (morning after or emergency or Levonorgestrel or hormon* or estrogen or oestrogen or progestogen) or |
| lactational adj2 (amenorrhea or amenorhoea) or |
| "Standard Days Method" or cycle beads or |
| unmet need or |
| met need |

Search 3: Delivery Care Terms

| exp childbirth/ or |
| --- |
| exp maternity services/ or |
| exp caesarean section/ or |
| exp parturition/ or |
| delivery adj2 (child or obstetric) or |
| caesarean or cesarean or c-section or csection or |
| birth* adj2 (home or facility or child) or childbirth* or |
| maternal or maternity servic* |

Search 4: Private Sector Terms

| exp private sector/ or |
| --- |
| private adj2 (sector or for-profit or facilit* or provider* or clinic* or hospital* or pharmac* or drug seller*) or privati#ed or privati#ation or public-private or private or |
| NGO or non-government* or nonprofit or not#for#profit or non#profit or non#for#profit or |
| informal adj2 (sector or provider*) or traditional adj2 (healer* or doctor* or provider*) or |
| charit* or FBO or mission or faith#based or religious or faith-inspired or Christian or Catholic or muslim or Islam* or |
| social* market* or |
| franchis* or |
| exp private firms/ or |
| out or service* adj2 (contracting or contracted or contract) |

Search 5: Final Search

1 AND (2 or 3) AND 4

*Popline Search Strategy*

Search 1: Sub-Saharan Africa Terms

| AFRICA, SUB SAHARAN |
| --- |

Search 2: Family Planning Terms

| CONTRACEPTION or |
| --- |
| CONTRACEPTIVE USAGE or |
| CONTRACEPTIVE AVAILABILITY or |
| CONTRACEPTIVE PREVALENCE or |
| FAMILY PLANNING or |
| FAMILY PLANNING PROGRAMS or |
| "family planning" or "birth control" or contracepti* or |
| unmet need or |
| met need |

Search 3: Delivery Care Terms

| MATERNAL-CHILD HEALTH SERVICES or |
| --- |
| CESAREAN SECTION or |
| CHILDBIRTH or |
| "delivery service*" or "obstetric delivery" or "child delivery" or |
| "home birth*" or "child birth*" or "childbirth*" or "home birth*" or "facility birth*" |
| maternal or "maternity service*" or |
| caesarean or cesarean or c-section or csection |

Search 4: Private Sector Terms

| PRIVATE SECTOR or |
| --- |
| COMMERCIAL SECTOR or |
| PRIVATELY SPONSORED PROGRAMS or |
| BAREFOOT DOCTORS or |
| TRADITIONAL BIRTH ATTENDANTS or |
| NGO or non-government* or nonprofit or not-for-profit or non-profit or |
| "informal sector" or "informal provider*" or "traditional healer*" or "traditional doctor*" or "traditional provider" or |
| charity or FBO or mission or faith-based or faith based or religious or |
| social* market* or |
| franchis* or |
| "contract* out" or "contract* service*" |

Search 5: Final Search

1 AND (2 or 3) AND 4
