# Supplementary material for: Meeting need vs. sharing the market: a systematic review of methods to measure the use of private sector family planning and childbirth services in sub-Saharan Africa
Source: BMC Health Serv Res. 2018 Sep 10;18:699. doi: 10.1186/s12913-018-3514-y (PMC6131793; doi:10.1186/s12913-018-3514-y)
Supplement: Supplementary file 3 — Descriptive summary of studies included in review. (DOCX 46 kb) [file 12913_2018_3514_MOESM3_ESM.docx]

**S3 Table:** Descriptive summary of studies included in review

| **Reference** | **SSA countries included in analysis** | **Data source** | **Data collection date** | **Survey coverage** | **Study design** | **Sample inclusion criteria** | **Sector categories** |
| --- | --- | --- | --- | --- | --- | --- | --- |
| Adogu et al. (2014) | Nigeria | Household survey | Not stated | 2 local government areas in 1 state | Cross-sectional | Women with a child aged 0-59 months | (1) **Private hospital:** not defined (2) **Public health facility**: not defined (3) **Maternity homes:** not defined (4) **TBA:** not defined (5) **Others home**: not defined |
| Agha & Do (2008) | Ghana, Kenya | DHS | **Kenya**: 1989, 1993, 1998, 2003  **Ghana:** 1988, 1993, 1998, 2003 | Nationally representative | Repeated cross-sectional | Women 15-49 years, currently married or in union | (1) **Private:** private commercial hospitals/clinics, private doctors, pharmacies, shops/stores (2) **Public:** government hospitals/clinics, government health centers (3) **NGO & others:** NGOs & friends/relatives |
| Amin (1998) | Sierra Leone | Household survey | 1993 | 2 districts | Cross-sectional | Women with at least one live birth in past 5 years | (1)  **Planned Parenthood clinics:** not defined (2) **Hospitals and public health units:** not defined (3) **MCH AID:** not defined |
| Aremu (2013) | Nigeria | DHS | 2008 | Nationally representative | Cross-sectional | Women 15-49 years, ever married, current users of modern contraception | (1) **Private:** private clinics and hospitals owned by an individual, non-government or religious organization, pharmacy stores, patent medicine sellers, hawkers (2) **Public:** any healthcare facility maintained by government at local, state, and national levels (3) **Informal:** friends, family, and other sources |
| Ayad et al. (1994) | 11 SSA countries | DHS | 1986-1990 | Nationally representative | Cross-sectional | Women 15-49 years, currently married or in union | (1) **Private pharmacy:** privately owned pharmacies (2) **Other private:** private organizations run by NGOs as well as private doctors, clinics, and other medical providers (3) **Government stationary**: any government-run facility at a fixed location (4) **Government mobile:** government outreach workers or mobile units (5) **Other source:** family, friends, and inconsistent responses |
| Bazant et al. (2009) | Kenya | Nairobi Urban Health and Demographic Surveillance System | 2006 | 2 urban informal settlements | Cross-sectional | Women 15-49 years with a birth within two years prior to survey | (2) **Private:** Religiously affiliated/mission, for-profit, or nonprofit (1) Woman's home/TBA's home: locations with no skilled care (3) **Government:** facilities administered by Nairobi City Council & Ministry of Health |
| Bell et al. (2003) | Ghana, Malawi | DHS | **Ghana:** 1988, 1993, 1998  **Malawi**: 1992, 2000 | Nationally representative | Cross-sectional | All women aged 15-49 years with live birth in the past 3 years | (1) **Private hospital/health center:** not defined (2) **Government hospital:** not defined (3) **Government health center:** not defined (4) **Other health facility:** government health posts, maternity facilities, private health centers (5) **Domiciliary:** home |
| Benova et al. (2015) | 30 countries representing 83% of SSA population | DHS | 2000-2013 | Nationally representative | Cross-sectional | Women 15-49 years with a birth in survey recall period | (1) **Private:** All births occuring in a facility outside of the public sector or with a private health professional (2) **Public sector:** public, government, or social security facilities (3) **Unclassifiable sector:** births occurring in a location that could not be classified as public or private |
| Berman & Rose (1996) | Botswana, Kenya, Sudan, Uganda | DHS | 1988-1990 | Nationally representative | Cross-sectional | Ever married women aged 15-49 years | (1) **Private:** Not defined (2) **Public:** Not defined (3) **Other:** Includes traditional providers, schools, churches, family, and friends |
| Brugha et al. (2003) | Kenya | DHS | 1998 | Nationally representative | Cross-sectional | Not stated | (1) **Private facility**: not defined (2) **Public facility**: not defined (3) **Home**: not defined |
| Campbell et al. (2015) | 30 countries representing 83% of SSA population | DHS | 2000-2013 | Nationally representative | Cross-sectional | Women 15-49 years, in need of or currently using modern contraception | (1) **Private sector**: all private providers, including private medical, private specialized drug sellers, private retailers, FBOs, and NGOs (2) **Public sector:** all government/public service locations, including public medical and non-medical sources (3) **Not classifiable**: reported missing source location or from husband, friend, relatives, other providers, or providers abroad |
| Campbell et al. (2016) | 30 SSA countries | DHS | 2004-2012 | Nationally representative | Cross-sectional | Women aged 15-49 years | (1) **Private sector**: all private providers, including private medical, private FBOs, NGOs, shops, pharmacies, drug sellers, and nightclubs (2) **Public sector:** all government/public service locations, including hospitals, polyclinics, doctors' offices, women's health centers, etc... (3) **Not classifiable**: reported missing source location or from husband, friend, relatives, other providers, or providers abroad |
| Chakraborty et al. (2016)* | Kenya | Household survey | 2013 | Not stated | Cross-sectional | Women aged 15-49 years who are sexually active | (1) **Franchise clinic:** facility that belongs to a network and is operated by a private sector actor (2) **Other private**: not defined (3) **Public facility:** public sector dispensary, health center, medical clinic, or sub-district hospital |
| Chapman et al. (2012) | 11 SSA countries | DHS | 1998-2008 | Nationally representative | Repeated cross-sectional | Women and men with non-marital, non-cohabiting partner | (1) **Private:** private hospital, doctor, other private, mission facility, other retail (2) **Pharmacy:** not defined (3) **Shop:** gas station or general shop (4) **Friends or family**: not defined (5) **Other**: bars, clubs, church |
| Delamou et al.(2014) | Guinea | DHS | 1999, 2005 | Nationally representative | Repeated cross-sectional | Not stated | (1) **Private medical sector:** clinics, pharmacies, NGOs/specialized associations (2) **Private not medical sector**: shops, kiosks bars (3) **Public facility**: not defined (4) **Other/unspecified:** not defined |
| Echoka et al. (2013) | Kenya | Facility data | 2010 | 1 district | Cross-sectional | All births | (1) **Private facility:** not defined (2) **Voluntary facility:** not defined (3) **Government facility:** not defined |
| Egede et al. (2015) | Nigeria | Survey with respondents recruited from a market | Not stated | 1 city/2 local government areas | Cross-sectional | Women aged 14-49 currently using any method of contraception, sexually active, and pre-menopausal | (1) **Private hospital:** not defined(2) **Patent medicine dealer:** not defined(3) **Open market:** not defined(4) **Family planning clinic:** not defined(5) **Public hospital:** not defined |
| Fotso et al. (2013) | Kenya | DHS | 1993, 1998, 2003, 2008/9 | Nationally representative | Repeated cross-sectional | Women currently married | (1) **Private/other**: includes NGOs and FBOs (2) **Public:** not defined |
| Ganle et al. (2014) | Ghana | Ghana Maternal Health Survey | 2007 | Nationally representative | Cross-sectional | Women 15-49 years | (1) **Private health facility:** not defined (2) **Public health facility**: not defined (3) **Home**: not defined (4) **Other**: not defined (5) **Missing**: not defined |
| Hodgkin (1996) | Kenya | Household survey | 1989 | 1 district | Cross-sectional | All households with at least 1 delivery in the past year | (1) **Private hospital or health center:** not defined (2) **Missionary hospital or health center:** not defined (3) **Government hospital or health center:** not defined (4) **Informal setting:** TBA's place, at home with TBA, at home without TBA, or other location |
| Hopstock et al. (1997) | 26 SSA countries | DHS | 1986-1996 | Nationally representative | Cross-sectional | Married women of reproductive age | (1) **Commercial:** for-profit providers, including pharmacies, doctors, nurses/midwives, shops/markets, traditional providers, and workplaces (2) **Nonprofit:** non-government owned providers that receive external funding, including clinics, mission facilities, non-governmental organizations, community-based distributors, and churches (3) **Public:** government-owned providers, institutions, fieldworkers, and schools (4) **Other:** friends and acquaintances, relatives, spouses, other, don't know/missing |
| Hotchkiss et al. (2011) | Nigeria, Uganda | DHS | **Nigeria:** 1999, 2003, 2008  **Uganda**: 1988, 1995, 2001, 2006 | Nationally representative | Repeated cross-sectional | Women 15-49 years, currently married or in union | (1) **Private commercial sector**: commercial outlets that sell contraceptive supplies and services, including chemists, shops, pharmacies, traditional healer/doctor, midwife, and private health facilities and workers (2) **Government sector**: not defined (3) **Other sources:** NGOs, FBOs, relatives, friends, others |
| Ikeako et al. (2006) | Nigeria | Household survey | 2004 | 1 city | Cross-sectional | Women who had a delivery in the last 3 months | (1) **Private-obstetrician-run hospitals:** hospitals managed by qualified obstetricians (2) **Teaching hospital/state specialist hospital:** hospital managed by qualified obstetricians, resident doctors, or medical officers with facilities for blood transfusion (3) **General hospital/private hospitals/mission hospitals:** hospital with general duty medical officer (but no specialist obstetrician) and facilities for operative deliveries (4) **Maternity homes/primary health centers:** homes/centers operated by state registered nurses/midwives without the assistance of doctors (5) **Traditional birth attendants:** as defined by WHO, 1992; not trained to handle complications and not registered or licensed to practice (6) **Spiritual houses:** churches, prayer houses, healing homes (7) **Home delivery:** conducted in a woman's home |
| Iyaniwura & Yussuf (2009) | Nigeria | Household survey | 2005 | 1 town | Cross-sectional | Women of reproductive age who carried at least 1 pregnancy to term in the past 5 years | (1) **Private hospital:** Not defined (2) **Government facility:** Not defined (3) **Home:** Not defined  (4) **Spiritual home:** Not defined (5) **Traditional/herbal home:** Not defined |
| Johnson et al. (2009) | Ghana | DHS | 1998, 2003 | Nationally representative | Repeated cross-sectional | Women who had a birth in the past 5 years | (1) **Private institution:** not defined (2) **Public institution**: not defined (3) **Home**: occurred under the supervision of untrained birth attendants, including SBAs |
| Khan et al. (2007) | 18 SSA countries | DHS | 1987-2004 | Nationally representative | Repeated cross-sectional | Women aged 15-49 years, currently married or in union | (1) **Private medical sector:** not defined (2) **NGOs:** not defined (3) **Public sector**: not defined (4) **Other sources:** not clearly defined, but includes shops, churches, and friends. NGOs included as "other" in time trend analysis |
| Kruk et al. (2009) | Tanzania | Household survey | 2007 | 1 district (excluding main town) | Cross-sectional | Women aged 18 years or above who delivered in the past 5 years | (1) **Mission health facility:** mission dispensary, health center, or hospital (2) **Government dispensary:** not defined (3) **Government health center:** not defined (4) **Government hospital:** not defined (5) **Home:** not defined (6) **On the way to a health facility:** not defined |
| Lafort et al. (2016) | Mozambique | Respondent-driven sampling survey | 2013-2014 | 1 city | Cross-sectional | Female sex workers | (1) **Private clinic:** Not defined (2) **Night clinic (NGO):** a sexual and reproductive health clinic targeting most-at-risk populations open during the evenings and operated by a non-governmental organization (3) **Informal health sector:** Not defined (4) **Public health facilities:** Not defined (5) **Community outreach:** Not defined (6) **Outside catchment area:** Not defined |
| Lewis & Kenney (1988) | Kenya, Liberia, Senegal, Zaire, Zimbabwe | Contraceptive Prevalence Surveys, DHS, other household survey | 1984-1986 | Nationally representative (?) | Cross-sectional | Not stated | (1) **Commercial:** for-profit hospitals, clinics, dispensaries, pharmacies, shops, traditional healers (2) **NGO:** nonprofit, non-governmental providers including religious groups and other charitable organizations (3) **Government:** government-owned facilities (4) **Other:** unspecified source, possibly including NGOs or other private sources when not included as a response option on the survey |
| Limwattananon et al. (2011) | 19 SSA countries | DHS | 1995-2006 | Nationally representative | Repeated cross-sectional | Women with at least one delivery in survey recall period | (1) **Private institution:** private for-profit hospitals, clinics, maternity homes; NGO & not-for-profit hospitals/clinics; mission hospitals/clinics, and other private facilities (2) **Public institution:** government hospital, health center/post, maternity home, or dispensary; community health center, primary health center, or other public facility (3) **Non-institutional:** home of TBA, midwife, relative, or pregnant woman; other non-facility |
| Matshidze et al. (1998) | South Africa | Facility data | 1990 | 1 metropolitan area | Cross-sectional | All births that occurred in a health facility during study data collection period | (1) **Private facilities:** not defined (2) **Public facilities:** not defined |
| Measurement, Learning & Evaluation project et al. (2011) | Kenya | Household survey | 2010 | 5 cities/urban centers | Cross-sectional | All women aged 15-49 years | (1) **Private facilities:** private hospitals, clinics, and doctors, including NGOs and FBOs (2) **Pharmacists/chemists:** not defined (3) **Public facilities:** not defined (4) **Other:** shops, kiosks, worksite clinics, voluntary counseling & testing centers |
| Nguyen et al. (2011) | Ethiopia, Kenya, Malawi, Rwanda, Tanzania, Uganda | DHS | 1999-2006 | Nationally representative | Cross-sectional & repeated cross-sectional | Women 15-49 years using modern contraceptives | (1) **Private (facilities):** for-profit hospitals and clinics (2) **Private (informal):** for-profit pharmacies and drug vendors (3) **Private not for profit:** non-governmental and faith-based providers (4) **Public:** not defined (5) **Other:** not defined |
| Nketiah-Amponsah and Arthur (2013) | Ghana | DHS | 2008 | Nationally representative | Cross-sectional | Women 15-49 years, "expectant mothers" | (1) **Private facility:** not defined (2) **Public facility:** delivered in public sector allopathic facility (3) **Home:** delivered at home without professional assistance |
| O'Meara et al. (2015) | Kenya | Household survey | 2011-2012 | 4 districts | Cross-sectional | Women aged 18 years or above who delivered in the past 5 years | (1) **Private clinic:** not defined (2) **Hospital/Nursing home:** not defined (3) **Health center/dispensary:** not defined (4) **Home:** not defined |
| Obare et al. (2014) | Kenya | Household survey | 2010-2012 | 7 districts | Repeated cross-sectional | Women aged 15-49 years who gave birth in the past 12 months or pregnant | (1) **Private facility:** not defined (2) **Public facility:** not defined (3) **Home:** not defined (4) **Other:** includes births on the way to health facility |
| Obare et al. (2015) | Kenya | Household survey | 2010-2012 | 7 districts | Repeated cross-sectional | Women aged 15-49 years who gave birth in the past 12 months or pregnant | (1) **Private facility:** not defined (2) **Public facility:** not defined (3) **Home/Other/Missing (delivery care only):** not defined (4) **Other/Missing (FP only):** not defined |
| Olusanya et al. (2010) | Nigeria | Community survey recruited at health facility | 2005-2008 | Participants recruited from BCG clinics at four health centers in Lagos | Cross-sectional | Women who delivered in a hospital | (1) **Private hospital:** not defined (2) **Public hospital:** not defined |
| Onwujekwe et al. (2013) | Nigeria | Household survey | 2010 | 6 states purposively selected from the 6 geopolitical zones | Cross-sectional | Female primary caregiver of childbearing age OR other woman of childbearing age OR male head of household | (1) **Private hospitals:** not defined (2) **Patent medical vendors:** not defined (3) **Pharmacy shops:** not defined (4) **Public hospitals:** not defined (5) **PHC:** not defined (6) **Others:** not defined |
| Osubor et al. (2006) | Nigeria | Household survey | 1999 | 1 rural community | Cross-sectional | Women aged 15-49 years who delivered in the past 1 year | (1) **Private clinic:** private maternity center, often owned by retired midwives (2) **Government clinic:** government-owned primary health care facility (3) **Traditional birth attendants:** members of the community who provided health services to pregnant women, informally trained |
| Oye-Adeniran et al. (2005) | Nigeria | Household survey | Not stated | 8 local government areas in 4 randomly selected states | Cross-sectional | Women aged 15-49 | (1) **Private clinic/hospital:** not defined (2) **Chemist/patent medicine shop:** not defined (3) **Market**: not defined  (4) **Roadside vendor/kiosk**: not defined  (5) **Drug peddler**: not defined  (6) **Pharmacy**: not defined  (7) **General hospital**: not defined  (8) **Health center**: not defined  (9) **Nursing/maternity homes**: not defined  (10) **Others**: not defined |
| Oye-Adeniran et al. (2006) | Nigeria | Household survey | 2002 | 4 states | Cross-sectional | Women aged 15-49 years | (1) **Chemists/patent medicine shops**: not defined (2) **Health centers:** not defined (3) **Family planning centers:** not defined (4) **General hospitals:** not defined |
| Rosen and Conly (1999) | 28 SSA countries | DHS | 1987-1998 | Nationally representative | Cross-sectional | Women 15-49 years, currently married or in union | (1) **Private commercial sector:** for-profit clinics, practitioners, and retail outlets (2) **Private non-profit sector:** not defined (3) **Public sector:** not defined |
| Ross et al. (2005) | 31 SSA countries | DHS | 1986-2003 | Nationally representative | Cross-sectional | Not stated | (1) **Private medical:** not defined (2) **Other private:** not defined (3) **Public:** not defined (4) **Other:** not defined |
| Sidze et al. (2014) | Senegal | Household survey | 2011 | 6 cities | Cross-sectional | Women aged 15-29 years | (1) **Private sector:** private hospitals, clinics, and other private sources (2) **Private hospital/clinic:** not defined (3) **Other private:** includes workplace clinics, youth centers, voluntary counseling and testing centers, shops, markets, and peer educators (4) **NGO/other:** not defined (5) **Public sector:** public hospitals, health centers, health posts, other public |
| Tabatabai et al. (2014) | Tanzania | Facility data | 2008 | All 16 hospitals in 12 purposively selected districts | Cross-sectional | All normal deliveries and c-sections that were recorded in districts with access to public and FBO hospitals from January - December 2008 | (1) **Faith-based organization hospitals**: faith-based not-for-profit private sector hospitals (2) **Public hospitals:** not defined |
| Ugaz et al. (2015) | 18 SSA countries | DHS & RHS | 1992-2012 | Nationally representative | Repeated cross-sectional | Women 15-49 years, currently married or in union | (1) **Private sector:** private clinics, private hospitals, private doctors, private pharmacies, and non-governmental organization facilities (2) **Public sector**: government clinics, government hospitals, government health centers, public family planning clinics, social security programs, public field workers (3) **Other sources**: shops, churches, friends, others |
| Waiswa et al. (2015) | Uganda | DSS | Baseline: 2007 Endline: 2011 | 2 districts | Cross-sectional | Baseline: Women with infants aged 1-4 months  Endline: Women of childbearing age with live birth in past 12 months | (1) **Private facilities:** not defined (2) **Public facilities:** not defined |
| Wang et al. (2012) | Kenya, Rwanda, Tanzania, Uganda | DHS | 2003-2010 | Nationally representative | Cross-sectional & repeated cross-sectional | All women aged 15-49 years | (1) **Private hospital/clinic:** not defined (2) **Private pharmacy:** not defined (3) **Other private:** not defined (4) **Shop:** not defined (5) **Friends/church:** not defined (6) **Public hospital:** not defined (7) **Public health center:** not defined (8) **Public clinic/dispensary:** not defined (9) **Other or missing:** not defined |
| White & Corker | Mali, Uganda | Household survey | 2013-2014 | Nationally representative | Cross-sectional | Not stated | (1) **Private/NGO sector:** not defined (2) **Public sector:** not defined (3) **Other sector/missing:** not defined |
| Winfrey et al. (2000) | 17-21 SSA countries | DHS | 1988-1994 | Nationally representative | Cross-sectional | Not stated | (1) **Commercial:** private sector pharmacies, shops, doctors, midwives, hospitals, clinics (2) **NGO:** subsidized private sector providers (3) **Public:** not defined (4) **Social Security:** government-organized insurance schemes (5) **Other:** not defined |
| Wodon et al. (2012) | 36 SSA countries | DHS | 1987-2008/9 | Nationally representative | Cross-sectional | Family planning: current users of any family planning method  Delivery care: Not stated | (1) **Private medical:** private secular and faith-inspired hospitals, clinics, pharmacies, doctors, mobile clinics, fieldworkers, other clinics, maternity homes, and other private medical care (2) **Public:** government hospitals, clinics, health posts, mobile clinics, fieldworkers, and other public providers (3) **Other:** Shops, markets, traditional practitioners, drug peddlers |
| Yoong et al. (2010) | 34 SSA countries | DHS | 1995-2008 | Nationally representative | Cross-sectional | All live births | (1) **Private health facility:** for-profit or non-profit/mission hospitals, clinics, health centers (2) **Public health facility**: public hospitals, clinics, health centers |
