# Supplementary material for: Meeting need vs. sharing the market: a systematic review of methods to measure the use of private sector family planning and childbirth services in sub-Saharan Africa
Source: BMC Health Serv Res. 2018 Sep 10;18:699. doi: 10.1186/s12913-018-3514-y (PMC6131793; doi:10.1186/s12913-018-3514-y)
Supplement: Supplementary file 4 — Studies on private sector family planning or childbirth care use by country & period. (PDF 257 kb) [file 12913_2018_3514_MOESM4_ESM.pdf]

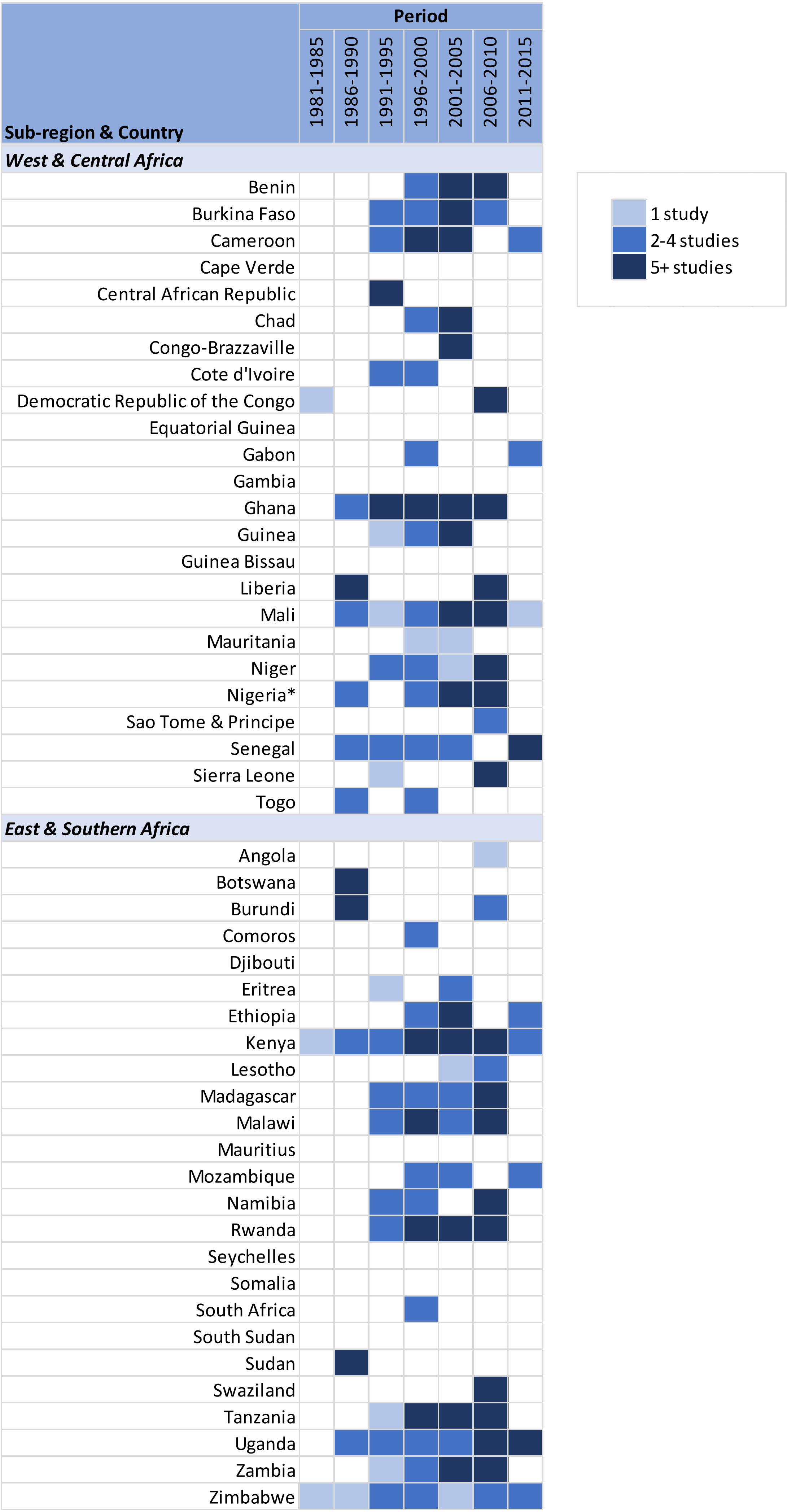

*Two studies excluded because authors omitted the dates of data collection.
