# Supplementary figures and images for: Meeting need vs. sharing the market: a systematic review of methods to measure the use of private sector family planning and childbirth services in sub-Saharan Africa
Source: BMC Health Serv Res. 2018 Sep 10;18:699. doi: 10.1186/s12913-018-3514-y (PMC6131793; doi:10.1186/s12913-018-3514-y)

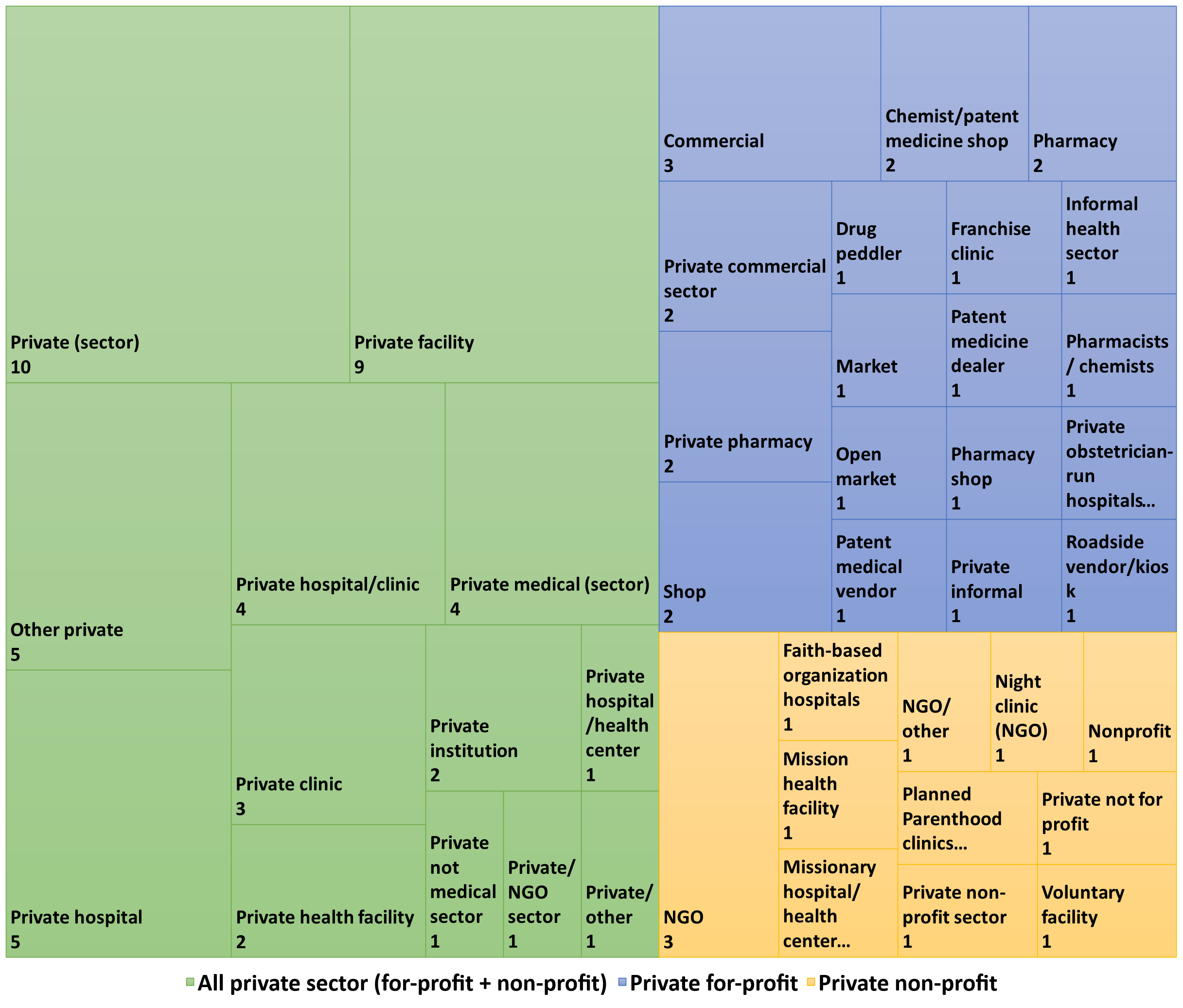

Supplement: Supplementary file 5 — Frequency of terms used to describe private sector sources in included studies. (PNG 336 kb) [file 12913_2018_3514_MOESM5_ESM.png]
