# Supplementary material for: Meeting need vs. sharing the market: a systematic review of methods to measure the use of private sector family planning and childbirth services in sub-Saharan Africa
Source: BMC Health Serv Res. 2018 Sep 10;18:699. doi: 10.1186/s12913-018-3514-y (PMC6131793; doi:10.1186/s12913-018-3514-y)
Supplement: Supplementary file 6 — Included study methods and results summary. (DOCX 121 kb) [file 12913_2018_3514_MOESM6_ESM.docx]

**S6 Table:**

**Family planning market share**

| **Reference** | **Unit of analysis** | **Outcome** | **Private sector market share estimate(s)** | **Numerator** | **Denominator** | **Treatment of missing information (source of care)** |
| --- | --- | --- | --- | --- | --- | --- |
| Agha & Do (2008) | Most recent FP supply | Private commercial sector market share for modern methods | **Kenya** 9.2% (1989) - 32.2% (2003)  **Ghana**  24.5% (1988) - 42.4% (2003) | Number of women who most recently received their FP method from private source | Current users of modern contraception aged 15-49 years, married or in union | Not stated |
| Amin (1998) | Not stated | Planned parenthood market share for contraceptive users | 20.8% | Number of women who obtained method from Planned Parenthood | Current users of any FP aged 12-49 years | Not stated |
| Aremu (2013) | Not stated | Private sector market share for modern methods | 55.30% | Number of women who received their FP method from private source | Current users of modern contraception aged 15-49 years, ever married (?) | Not stated |
| Ayad et al. (1994) | Most recent FP supply | Private sector market share for: (a) all modern methods; (b) clinical vs. supply methods; (c) individual methods | 2% (Burundi) - 63% (Liberia) | Number of women who most recently received their FP method by source | Current users of modern contraception aged 15-49 years, married or in union | "Don't know" and missing responses excluded from analysis |
| Berman & Rose (1996) | Not stated | Private market share for contraceptive users | **Botswana** 7.3% **Kenya** 27.8% **Sudan**  35.9% **Uganda** 44.1% | Number of women who obtained method from private sector source | "Reported use" | Not stated |
| Campbell et al. (2015) | Most recent FP supply | Private sector market share for modern methods among (a) all users, (b) users with classifiable source | (a) 35% (b) 38% | Number of women who most recently obtained their modern FP method from a private sector source | All women aged 15-49 years who are currently using modern FP from a source with a classifiable sector | Provided estimates including and excluding women with missing information on sector of care |
| Campbell et al. (2016) | Not stated | Private market share for: (a) Users of modern methods (b) Users of appropriate delivery care | (a) Overall: 38%; Range (6% - 80%) (b) Overall: 22%; Range (0% - 77%) | (a) Number of women who obtained method from private sector source (b) Number of women who delivered their most recent child in the private sector | (a) All women aged 15-49 years who are currently using modern FP from a source with a classifiable sector (b) All women 15-49 years who used appropriate care from a classifiable sector | Women with source of care whose sector or location of care could not be classified were excluded from analysis |
| Chakraborty et al. (2016) | Not stated | (a) Franchised clinic market share for current FP users (b) Other private market share for current FP users | (a) 1.58% - 16.78% (b) 20.12% - 27.89% | Number of women who obtained their method from a private source | Current users of any FP, aged 15-49 years and sexually active | Not stated |
| Chapman et al. (2012) | Most recent condom supply | (a) Private market share for condoms (b) Pharmacy market share for condoms (c) Shop market share for condoms | **MEN (a)** T1: 0.1% (Cameroon, 1998) - 17.6% (Malawi, 2000) T2: 0.7% (Guinea, 2005) - 11.6% (Uganda, 2006)  **(b)** T1: 0.1% (Malawi, 2000) - 36.9% (Guinea, 1999) T2: 0.1% (Malawi, 2004) - 13.6% (Mali, 2006)  **(c)** T1: 9.3% (Namibia, 2000) - 83.3% (Benin, 2001) T2: 40.4% (Zambia, 2007) - 70.7% (Kenya, 2003)  **WOMEN (a)** T1: 0.7% (Cameroon, 1998) - 16.0% (Malawi, 2000) T2: 0.9% (Namibia, 2000) - 15.8% (Uganda, 2006)  **(b)** T1: 0.0% (Malawi, 2000) - 26.5% (Cameroon, 1998) T2: 0.1% (Malawi, 2004) - 26.0% (Mali, 2006) **(c)** T1: 20.6% (Namibia, 2000) - 72.9% (Benin, 2001) T2: 31.3% (Zambia, 2007) - 67.4% (Cameroon, 2004) | Number of wo(men) who obtained their condom from a private source | Wo(men) who used a condom at last intercourse with non-marital, non-cohabiting partner | Not stated |
| Delamou et al.(2014) | Not stated | Private sector market share for (a) oral contraceptives, (b) injectables, (c) condoms | 1999 - 2005 (a) 43.6% - 46.6% (b) 17.6% - 10.1% (c) 83.1% - 46.6% | Number of women who obtained method from a private medical or not medical sector source | not stated | Included in other/unspecified category |
| Egede et al. (2015) | Not stated | (a) Private hospital market share for modern FP (b) Patent medicine dealer market share for modern FP (c) Open market market share for modern FP | (a) 13% (b) 51% (c) 5% | Number of women who received FP from a private sector source | Current users of modern FP | Not stated |
| Fotso et al. (2013) | Not stated | Private/other sector market share for modern methods | 1993: 43.5% 1998: 47.4% 2003: 55.1% | Number of women who received modern FP from private/other source | Current users of modern contraception, currently married | Not stated |
| Hopstock et al. (1997) | Most recent FP supply | n/a | n/a | n/a | n/a | n/a |
| Hotchkiss et al. (2011) | Most recent FP supply | Private commercial share for modern methods | **Nigeria**  1999: ~35% 2008: ~59%  **Uganda** 1989: ~10% 2006: ~55% | Number of women who most recently obtained method from a private commercial source | Current users of modern contraception aged 15-49 years, married or in union | Not stated |
| Khan et al. (2007) | Most recent FP supply | (a) Private medical market share for modern methods (b) NGO market share for modern methods | (a)  Lowest: 8%, Burkina Faso, 2003 Highest: 57%, Nigeria, 2003  (b) Lowest: 0%, many countries Highest: 12%, Malawi, 2000  How to show changes over time for multple countries? | Number of women who most recently obtained method from a private medical or NGO source | Current users of modern contraception aged 15-49 years, married or in union | Not stated |
| Lafort et al. (2016) | Most recent FP supply | (a) Private clinic market share for FP (b) Night clinic/NGO market share for FP (c) Informal health sector market share for FP | (a) 0.8% (b) 30.1% (c) 15.1% | Number of women who received FP from a private sector source | Current users of any FP | Not stated |
| Lewis & Kenney (1988) | Not stated | (a) Commercial market share for all (modern & traditional) FP methods (b) NGO market share for all (modern & traditional) FP methods | **Kenya** (a) 8.4% (b) 32.2% **Liberia** (a) 18.3% (b) 48.2% **Senegal** (a) 50.0% (b) -- **Zaire** (a) 28.7% (b) 3.6% **Zimbabwe** (a) 9.2% (b) 46.2% | Number of women who received FP from a private sector source | Current users of any FP | Not stated |
| Measurement, Learning & Evaluation project et al. (2011) | Most recent FP supply | (a) Private facility market share for modern methods (b) Pharmacy/chemist market share for modern methods | (a) ~10% (Kakamega) - 44% (Mombasa)  (b) 11% Kakamega - 25% (Nairobi) | Number of women who most recently obtained method from a private sector source | Current users of modern contraception, aged 15-49 years | Not stated |
| Nguyen et al. (2011) | Not stated | (a) Private for profit (facilities) market share for modern methods (b) Private for profit (pharmacies) market share for modern methods (c) Private not for profit market share for modern methods | Exact figures not given --displayed in bar chart; also gives private market share for pills, IUDs, and condoms separately | Number of women who obtained method from private sector source | Current users of modern contraception aged 15-49 years | Not stated |
| Obare et al. (2015) | Most recent FP supply | Private facility market share for any FP | 2010/11: 14.4% - 15.6% 2012: 70.7% - 71.4% | Number of women who received FP from private facility | All women who used FP in the past 12 months | Including in other sector/missing category |
| Onwujekwe et al. (2013) | Not stated | Market share for individual methods | Oddly defined/calculated | Number of individuals that received method from private hospital, pharmacy, or patent medical vendor | Not clear | Not stated |
| Oye-Adeniran et al. (2005) | Not stated | (a) Private clinic/hospital market share for all FP methods (b) Chemist/patent medicine shop (c) Market vendor market share for all FP methods (d) Roadside vendor/kiosk market share for all FP methods (e) Drug peddler market share for all FP methods | (a) 10.2% (b) 19.7% (c) 4.5% (d) 0.8% (e) 0.4% | Number of women who procured their method from sources (a)-(e) | Current users of any FP, 15-49 years | Not stated |
| Oye-Adeniran et al. (2006) | First FP supply | Chemists/patent medicine shop market share for FP | 16.40% | Number of women who received FP from a chemist/patent medicine shop | Women who had ever used contraception (?) | Women without information on first source of care were excluded from analysis |
| Rosen and Conly (1999) | Not stated | Private commercial share for modern methods | 27% 0.3% (Rwanda, 1992) - 68% (Cote d'Ivoire, 1994) | Number of women using modern contraception from a private commercial source | Current users of modern contraception aged 15-49 years, married or in union | Not stated |
| Ross et al. (2005) | Not stated | (a) Private medical market share for modern methods (b) Other private market share for modern methods | (a) 1.2% (Burundi, 1987) - 53.9% (Liberia, 1986) (2) 0.0% (Eritrea, 2002) - 35.8% (Togo, 1998) | Number of women using modern contraception from a private medical or other private source | Current users of modern contraception | Included in the denominator |
| Sidze et al. (2014) | Most recent FP supply | (a) Private (commercial) sector market share for modern methods (b) Private hospital/clinic market share for modern methods (c) Other private market share for modern methods (d) NGO/other market share for modern methods | (a) 26.1% (b) 8.2% (c) 17.9% (d) 7.4% | Number of women who received FP from a private sector source | Current users of modern contraception, aged 15-29 years | Not stated |
| Ugaz et al. (2015) | Not stated | (a) Private medical sector market share for modern methods (b) Private medical sector market share for long acting/permanent methods (c) Private medical sector market share for short-acting methods | **1992-2000** (a) Overall: 27% (b) Overall: 1.2%; Range:   **1998-2006**  Overall: 30% Range:  **2005-2012** Overall: 28% Range: | Number of women using modern contraception from a private sector source | Current users of modern contraception aged 15-49 years, married or in union | Not stated |
| Wang et al. (2012) | Most recent FP supply | (a) Private hospital/clinic (excl. condoms) (b) Private pharmacy market share for modern nethods (excl. condoms) (c) Other private market share for modern methods (excl. condoms) (d) Shop market share for modern methods (excl. condoms) | **Kenya (2003 & 2008/09)** (a) 27.1% (2008/09) - 36.6% (2003) (b) 5.3% (2003) - 9.2% (2008/09) (c) 0.6% (2008/09) - 1.9% (2003) (d) 0.1% (2008/09) - 0.2% (2003)  **Rwanda (2007/08)** (a) 3.0% (b) 0.5% (c) 2.2% (d) 0.0%  **Tanzania (2004/05)** (a) 9.6% (b) 5.9% (c) 4.0% (d) 0.4%  **Uganda (2006)** (a) 47.8% (b) 5.8% (c) 0.8% (d) 0.0% | Number of women who most recently obtained method from a private sector source | Current users of modern contraception (excl condoms) aged 15-49 years, married or in union | Included in other/missing category |
| White & Corker | Not stated | Private/NGO market share for IUDs | **Mali:** 21.7% **Uganda:** 50.4% | Not stated | not stated | Including in other sector/missing category |
| Winfrey et al. (2000) | Most recent FP supply | (a) Commercial market share for modern methods (b) NGO market share for modern methods | (a) ~5% (Niger) - 50% (Cameroon) (b) ~0.0% (Niger) - 50% (Liberia) | Number of women who obtained method from private sector source | Current users of modern contraception, married or in union | Not stated |
| Wodon et al. (2012) | Not stated | (a) Private medical sector market share for modern FP (b) Private medical sector market share for non-modern FP | (a) Simple average: 27.98%  1.78% (Sao Tome & Principe, 2008/9) - 61.16% (Nigeria, 2008) (b) Simple average: 9.24% 0.63% (Mozambique, 2003) - 33.45 (DRC, 2007) | Number of women who received FP from a private sector source | (a) Modern family planning users (b) Non-modern family planning users | Not stated |

**Childbirth service market share**

| **Reference** | **Unit of analysis** | **Outcome** | **Private sector market share estimate(s)** | **Numerator** | **Denominator** | **Treatment of missing information (source of care)** |
| --- | --- | --- | --- | --- | --- | --- |
| Benova et al. (2015) | Most recent birth | Private sector market share for delivery care among: (a) all women who used appropriate care (in facility or with SBA), (b) women who used appropriate care in classifiable sector | (a) 20% (b) 22% | Number of women who deliverd in a private sector source | (a) All women who used appropriate care (b) Women who used appropriate care from a classifiable sector | Provided estimates including and excluding women with missing information on sector of care |
| Campbell et al. (2016) | Most recent birth | Private market share for users of appropriate delivery care | Overall: 22%; Range (0% - 77%) | Number of women who delivered their most recent child in the private sector | All women 15-49 years who used appropriate care from a classifiable sector | Women with source of care whose sector or location of care could not be classified were excluded from analysis |
| Echoka et al. (2013) | All births captured in facility records during study period | (a) Private market share for facility births (b) Voluntary market share for facility births | (a) 10.0% (b) 2.8% | Number of births that occurred in a private or voluntary facility | All births recorded in facility records | Not stated |
| Matshidze et al. (1998) | All births captured in facility records during study period | Private market share for facility births | 15% | Number of women who delivered in private facility | All women who delivered in a facility | Not stated |
| Olusanya et al. (2010) | Not stated | Private market share for hospital births | 50.3% | Number of women who delivered in a private hospital | Women who sought care at selected BCG clinics and gave birth in a hospital | Not stated |
| Tabatabai et al. (2014) | All births captured in facility records during study period | (a) NGO market share for normal deliveries in hospitals (b) NGO market share for c-sections in hospitals | (a) 27.6% (b) 47.9% | Number of normal deliveries or c-sections that occurred in an FBO hospital | All births recorded in hospital records as belonging to women who delivered at a hospital in their home district | Not stated |
| Waiswa et al. (2015) | Not stated | Private sector market share for facility deliveries at endline | Baseline: 36% Endline: 22% | Number of women who delivered in a private facility | All women of childbearing age who delivered in a health facility | Not stated |

**Family planning coverage**

| **Reference** | **Unit of analysis** | **Outcome** | **Estimate** | **Need definition** | **Numerator** | **Denominator** | **Treatment of missing information** |
| --- | --- | --- | --- | --- | --- | --- | --- |
| Campbell et al. (2015) | Most recent FP supply | Private sector coverage of modern FP need | Overall: 14% 2.1% (Chad, 2004) - 29.5% (Swaziland, 2006/7) | (a) Using a modern method OR (b) Did not desire birth in next 2 years & married or have had sex in past 30 days AND (c) Not infecund or menopausal | Number of women who most recently obtained their modern FP method from a private sector source | All women aged 15-49 years in need of family planning | Women with missing information on FP need considered to not have FP need; no missing information on delivery need; women who sought care from unclassifiable source not included in private sector |
| Campbell et al. (2016) | Not stated | Private sector coverage of: (a) modern family planning need (b) delivery care need | (a) Overall: 14% (b) Overall: 10% | (a) see Campbell et al (2015) (b) All women with birth in survey recall period | (a) Number of women who obtained a modern method from a private sector source (b) Number of women who received appropriate delivery care in the private sector | (a) All women in need of FP (b) All women who gave birth in survey recall period | Women with missing information on FP need considered to not have FP need; no missing information on delivery need; women who sought care from unclassifiable source not included in private sector |
| Hopstock et al. (1997) | Most recent FP supply | Proportion of married women using a modern method from: (a) Commercial source (b) Nonprofit source | (a) 0.0% (Burundi, 1987) - 4.9% (Ghana, 1993) (b) 0.0% (Burundi, 1987; Niger, 1992; Rwanda, 1992) - 6.7% (Kenya, 1993) | All women aged 15-49 years, married or in union | Number of women using modern FP from a private source | All women aged 15-49 years, married or in union | Included in "other" category |
| Ugaz et al. (2015) | Not stated | Proportion of married women using:  (a) long acting/permanent modern FP from private sector source (b) short acting modern FP from private sector source | 1992-2000: (a) 0.8%, (b) 2.4% 1998-2006: (a) 0.9%, (b)3.9% 2005-2012: (a) 1.1%, (b) 4.6% | All women aged 15-49 years, married or in union | Number of women using modern FP from a private sector source | All women aged 15-49 years, married or in union | Not stated |
| Winfrey et al. (2000) | Most recent FP supply | Proportion of married women using a modern method from the commercial sector | 0.02% (Mali, 1987) - 5.36% (Zimbabwe,1994) | All women married or in union | Number of women using modern FP from a commercial source | All women married or in union | Not stated |

**Childbirth service coverage**

| **Reference** | **Unit of analysis** | **Outcome** | **Estimate** | **Need definition** | **Numerator** | **Denominator** | **Treatment of missing information** |
| --- | --- | --- | --- | --- | --- | --- | --- |
| Adogu et al. (2014) | Most recent birth | Private facility coverage of delivery care | 18.9% (rural) - 50.5% (urban) | All women with birth in past 5 years | Number of women who delivered in a private hospital | All women who gave birth in the past 5 years | Included in "no response" category |
| Bazant et al. (2009) | Not stated | Private sector coverage of delivery care | 45% | All women with birth in study recall period | Number of women who delivered in a private sector facility | All women aged 15-49 years who gave birth in recall period | Not stated |
| Bell et al. (2003) | All live births in study recall period | Private facility coverage of delivery care | **Ghana**  1988: unknown 1993: 5.1% 1998: 5.4%  **Malawi** 1992: 10.5% 2000: 10.5% | All live births that occurred within 3 years prior to survey | Number of births delivered in a private sector hospital or health center | All live births that occurred in the 3 years before the survey (multiples only counted once) | Not stated |
| Benova et al. (2015) | Most recent birth | Private sector coverage of delivery care | 10% | All women with birth in study recall period | Number of women who delivered in a private sector facility | All women aged 15-49 years who gave birth in recall period | Women whose source of care was missing or could not be classified were included in an "unclassifiable" category |
| Brugha et al. (2003) | Not stated | Private sector coverage of delivery care | 11.20% | Not stated | Number of women who delivered in a private sector facility | Not stated | Not stated |
| Campbell et al. (2016) | Most recent birth | Private sector coverage of delivery care need | Overall: 10% | All women with birth in survey recall period | Number of women who received appropriate delivery care in the private sector | All women who gave birth in survey recall period | Women with missing information on FP need considered to not have FP need; no missing information on delivery need; women who sought care from unclassifiable source not included in private sector |
| Echoka et al. (2013) | All births captured in facility records during study period | n/a | n/a | n/a | n/a | n/a | n/a |
| Ganle et al. (2014) | Most recent live birth or stillbirth | Private facility coverage of delivery care | 11% | All women with a live birth or stillbirth in the study recall period | Number of women who delivered in a private sector facility | All women aged 15-49 years with live birth or still birth in study recall period | Women whose source of care was missing were included in a "missing" category |
| Hodgkin (1996) | Most recent birth that occurred in a household | (a) Missionary hospital/health center coverage of delivery care  (b) Private hospital/health center coverage of delivery care | (a) 16.7% (b) 2.0% | All births that occurred within 1 year prior to the survey | Number of births that occurred in a private facility | All women who gave birth in the past 1 year | Only described for the covariates, not the outcome |
| Ikeako et al. (2006) | Most recent birth | Private obstetrician-run hospital coverage of delivery care | 17.7% | All births that occurred within 3 months prior to survey | Number of births delivered in private obstetrician-run hospital | All births that occurred in the 3 months before the survey | Not stated |
| Iyaniwura & Yussuf (2009) | Most recent birth | Private hospital coverage of delivery care | 24.5% | All births that occurred within 5 years prior to survey | Number of births delivered in a private hopsital | All births that occurred within 5 years prior to survey | Not stated |
| Johnson et al. (2009) | Most recent birth | Private institution coverage of delivery care | 1998: 11.4% 2003: 8.6% | All women with a birth in the study recall period | Number of women who delivered in a private institution | All women with a birth in study recall period | Not stated |
| Kruk et al. (2009) | Most recent birth | Mission health facility coverage of delivery care | 17.0% | All women with birth in past 5 years | Number of births that occurred in a mission facility | All women who gave birth in the past 5 years | Not stated |
| Limwattananon et al. (2011) | Woman- based definition, all births pooled and source of care determined by algorithm:  Public: At least one delivery in a public institution, regardless of where the other births occurred  Private (only): At least one delivery in a private institution, with no deliveries in a public institution  Non-institutional: All births occured in a non-institutional setting | Private institutional (only) coverage | Estimates difficult to ascertain from included figure | Women with at least one delivery in survey recall period | Number of women who fulfill criteria for "private institutional" category | All women with at least one delivery in survey recall period | Not stated |
| Matshidze et al. (1998) | All births captured in facility records during study period | n/a | n/a | n/a | n/a | n/a | n/a |
| Measurement, Learning & Evaluation project et al. (2011) | Most recent birth | Private facility coverage of delivery care | 17% (Kakamega) - 44% (Nairobi) | All women with birth in study recall period | Number of women who delivered in a private sector facility | All women aged 15-49 years who gave birth in recall period | Not stated |
| Nketiah-Amponsah and Arthur (2013) | Not stated | Proportion of expectant mothers who delivered in a private facility | No overall estimate given; only given for sub-groups | Not stated | Number of women who delivered in a private sector facility | All women aged 15-49 who are expectant mothers | Not stated |
| O'Meara et al. (2015) | Most recent birth | Private clinic coverage of delivery care | 0.5% - 5.2% | All women who gave birth within 5 years prior to survey | Number of births that occurred in a private clinic | All births that occurred in the 5 years before the survey | Not stated |
| Obare et al. (2014) | All births that occured in 2 years before survey | Private facility coverage of delivery care | 2010/11: 6.5% - 21.1% 2012: 13.2% - 29.9% | All births that occurred within 2 years prior to data collection | Number of births that occurred in a private facility | All births that occurred in the 2 years before the survey | Not stated |
| Obare et al. (2015) | Most recent live birth in 2 years before the survey | Private facility coverage of delivery care | 2010/11: 12.7% - 14.1% 2012: 13.0% - 21.9% | All women who gave birth within 2 years prior to data collection | Number of births that occurred in a private facility | All women with at least one delivery in 2 years before the survey | Included in home/missing/other category |
| Olusanya et al. (2010) | Not stated | n/a | n/a | n/a | n/a | n/a | n/a |
| Osubor et al. (2006) | Not stated | Private clinic coverage of delivery care | 49.4% | All women with birth in the past 1 year | Number of women who delivered in a private clinic | All women aged 15-49 who delivered in the past 1 year | Not stated |
| Tabatabai et al. (2014) | All births captured in facility records during study period | n/a | n/a | n/a | n/a | n/a | n/a |
| Waiswa et al. (2015) | Not stated | Private facility coverage of delivery care | Baseline: 25.1% Endline: 17.4% | Baseline: All women who have a child 1-4 months  Endline: All women of childbearing age with live birth in past 12 months | Number of women who delivered in a private sector facility | Baseline: All women who have a child 1-4 months  Endline: All women of childbearing age with live birth in past 12 months | Women whose source of care was missing were included in a "missing" category |
| Wodon et al. (2012) | Most recent birth | Private medical sector coverage of delivery care | Simple average: 6.78%  0.11% (Comoros, 1996) - 21.81% (DRC, 2007) | Most recent birth | Number of women who delivered in the private sector for their last birth | All women who gave birth (unspecified period) | Not stated |
| Yoong et al. (2010) | All live births in study recall period | Private facility coverage of delivery care | Simple average: 7.7% | All live births that occurred within 3 years prior to survey | Number of births delivered in a private sector facility | All live births that occurred in the 3 years before the survey | Not stated |
